# Halogenase-Assisted Alkyne/Aryl Bromide Sonogashira Coupling for Ribosomally Synthesized Peptides

**DOI:** 10.1021/jacs.4c12210

**Published:** 2024-10-23

**Authors:** Nirmal Saha, F. N. U. Vidya, Ramon Xie, Vinayak Agarwal

**Affiliations:** † School of Chemistry and Biochemistry, Georgia Institute of Technology, Atlanta, Georgia 30332, United States; ‡ School of Chemistry and Biochemistry, Georgia Institute of Technology, Atlanta, Georgia 30332, United States; § School of Biological Sciences, Georgia Institute of Technology, Atlanta, Georgia 30332, United States

## Abstract

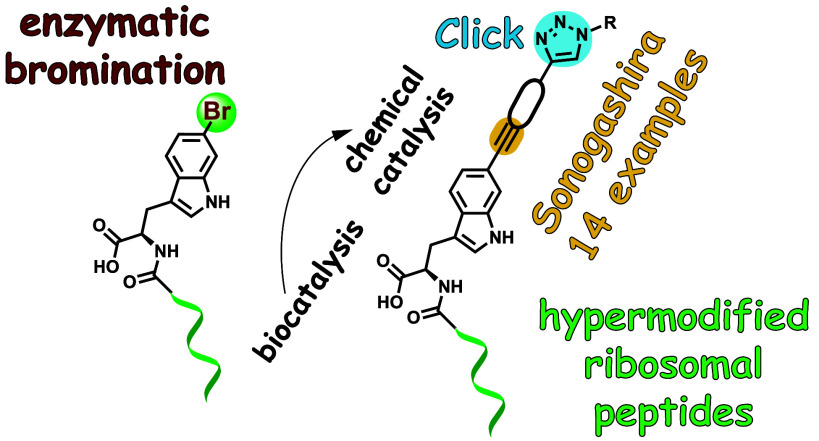

We describe the enzymatic bromination of ribosomally synthesized peptides and develop protocols for Sonogashira coupling of peptidic aryl bromides with a panel of alkynes. Using this workflow, entirely new chemical handles are introduced onto ribosomal peptides, including but not limited to terminal alkynes, which enable further diversification via alkyne–azide click chemistry. Regiospecific enzymatic installation of the aryl bromide circumvents genetic code expansion and passivation of other reactive handles on the peptide chain, representing the applicability of biocatalysts in peptide modification chemistry.

Chemical modification of peptides and proteins is essential to expand their structural and functional novelty and fully exploit their myriad of pharmaceutical applications. Various means to introduce reactive handles in polypeptides—beyond the ones available to the proteinogenic amino acids—have been developed.^1−3^ Among these are amino acid building blocks with halogenated side chains. While halogenation is one of the most versatile C–H functionalization strategies and is ubiquitous in chemical synthesis, regiospecific halogenation of peptides and proteins is outside the purview of chemical halogenation catalysts; the site for halogenation is *usually* governed by substrate reactivity rather than by the catalyst itself. Thus, to introduce halogens into peptides and proteins, entirely new strategies in chemical biology are used. Genetic code expansion allows for the incorporation of halogenated nonproteinogenic amino acids in polypeptide chains.^4^ Reactive side chains for proteinogenic amino acids, such as cysteine thiols, can be conjugated to halogen-bearing handles after passivation of other similarly reactive residues.^5^ The *de novo* synthesis of peptides and proteins with halogenated handles has also been realized.^6^ Taking a leaf out of the chemical synthesis handbook, each of these halogenation strategies has been combined with transition-metal-assisted chemical derivatization reactions, such as the Suzuki-Miyaura cross-coupling, to derivatize peptides and proteins (Figure 1A).

**Figure 1:**
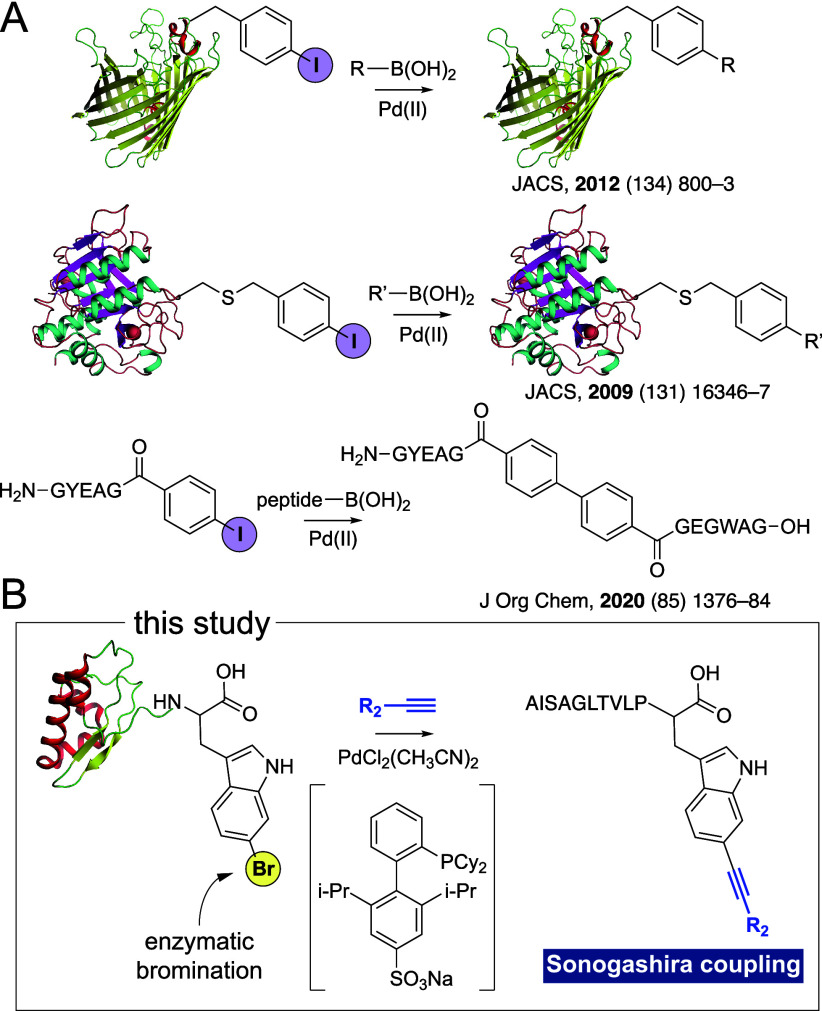
(A) Aryl iodide introduction into proteins by genetic code expansion (top), ligation to a side chain Cys thiol (middle), and preinstallation into synthetic peptides (bottom) enable Pd-catalyzed Suzuki-Miyaura cross-coupling reactions with boronic acids. (B) In this study, enzymatic regiospecific bromination of the C-terminal Trp side chain indole allows for Sonogashira coupling of alkynes with a ribosomally synthesized peptide.

Enzymes involved in the biosynthesis of ribosomally synthesized and post-translationally modified peptides (RiPPs) offer an alternate route for biocatalytic introduction of novel modifications and functionalities into peptides.^7^ While RiPP modification enzymes indeed suffer from poor atom economy (substrate recognition is dependent on an N-terminal leader region of the peptide that in itself is not modified and is dispensed with after the enzymatic transformation), they do offer exquisite regiocontrol. Case in point are the recently discovered RiPP halogenases. Of all RiPP halogenases and small molecule halogenases that accommodate peptidic substrates, the flavin-dependent RiPP halogenase SrpI brominates *only* the C-terminal Trp residue of the SrpE substrate peptide at the indole-6 position; other Trp residues in the peptide substrate are not modified.^8−10^ Furthermore, SrpI is strictly selective for bromination with no contaminating chlorination activity.^11,12^

In this study, we sought to determine the optimal substrate peptide sequence for SrpI and develop protocols for the copper-free Sonogashira coupling of terminal alkynes to SrpI-delivered brominated ribosomal peptides. As a tool for modifying amino acids, the Sonogashira cross-coupling reaction has found diverse applications such as in fluorescence tagging, biotinylation, and installing ^18^F handles for imaging.^13−15^ However, Sonogashira coupling for ribosomally derived peptides and proteins has typically involved the introduction of a peptide-alkyne or peptide-iodide using the chemical strategies illustrated in Figure 1A.^16,17^ In this study, we leverage the regiospecificity and obligate bromination activity of SrpI to generate peptidic aryl bromide substrates for Sonogashira coupling (Figure 1B). With a view toward palladium-assisted C–C bond forming reactions, bromination is particularly desirable because it opens up avenues for these reactions to proceed at milder conditions as compared to chlorinated substrates. Specifically, reaction temperatures required for Sonogashira coupling using aryl chlorides are prohibitive (>100 °C) for application to peptides and proteins.^18,19^ Hence, the bromination activity of SrpI provides a unique opportunity to interface a peptide-modifying biocatalyst with chemical methodology development. Peptide iodination using RiPP biosynthetic enzymes is currently out of reach.

Guided by binding to the substrate peptide’s N-terminal leader region, SrpI is permissive for diverse C-terminal core peptides as long as the Trp residue—the side chain of which is brominated at the indole-6 position—is positioned at the C-terminus.^20^ Though SrpI can (di)brominate a Tyr side chain phenoxyl, the physiological substrate is the Trp indole.^11^ Ribosomally synthesized core peptides ranging from 3 to 11 residues, appended to the SrpE leader, were tested as substrates for *in vitro* bromination by SrpI using the NAD(P)H-generating phosphite dehydrogenase PTDH and the flavin-reductase RebF as partner enzymes (Figure 2A, Table S1).^12^ While the core sequences of these susbtrate peptides are quite divergent from the native azoline-containing native substrates for SrpI, bromination still proceeds as long as the Trp residue is present at the C-terminus.^11,20^ Product yields were determined using liquid chromatography/mass spectrometry (LC/MS). In the core peptides used in this study, the Pro residue preceding the terminal Trp residue (which gets brominated by SrpI) provides a diagnostic MS^2^ fragmentation signature due to the labile nature of the prolyl amide bond allowing for any modifications on the Trp residue to be tracked (*vide infra*).^21^ Using a panel of substrate peptides with a conserved leader sequence but different core sequences, we discerned that the penta- and hexapeptides offered maximal productivity for SrpI, with the yield falling off at either end of the range (Figures 2B and S1–S15, Table S2). Product MS^2^ fragmentation spectra demonstrated that the C-terminal Trp was brominated (Figure 2C). Henceforth, the hexapeptide LTVLPW is used as the core peptide for bromination by SrpI. The *in vitro* bromination strategy developed herein allowed for large volume reactions. The core peptide was excised from the proteusin leader by proteolytic digestion by Glu-C to afford a undecapeptide brominated product in multimilligram scale (Figure S16). With the brominated undecapeptide in hand, we were well positioned to explore reaction conditions that afford Sonogashira coupling of alkynes to a ribosomally synthesized peptide.

**Figure 2:**
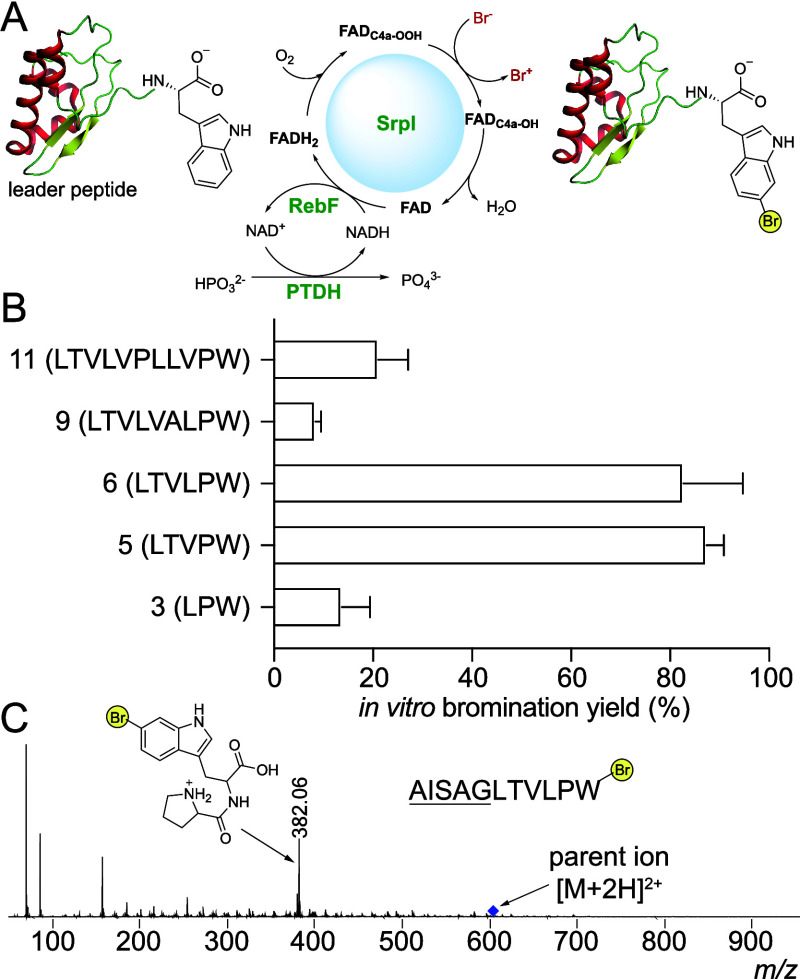
(A) Scheme for SrpI-catalyzed *in vitro* bromination. The leader peptide is shown in cartoon representation. (B) Product yields for different core peptides. Means and standard deviations from three independent reactions are plotted. All reactions were conducted under identical conditions (see Supporting Information for details). (C) MS^2^ fragmentation spectra corresponding to the brominated undecapeptide demonstrate the characteristic Pro-Trp product ion. The undecapeptide [M+2H]^2+^ parent ion is illustrated by the blue diamond. Digestion by endoproteinase Glu-C furnishes an undecapeptide product, of which the underlined five amino acids are derived from the SrpE leader.

Buchwald reported the development of the hydrophilic sulfonated ligand sXPhos for copper-free Sonogashira coupling of small molecule aryl bromides with alkynes using a water/acetonitrile biphasic solvent system and *bis*PdCl_2_(CH_3_CN)_2_ as the catalyst.^22^ Subsequently, Goss reported the use of this catalyst/ligand pair, and microwave/100 °C reaction conditions for Sonogashira coupling of alkynes to bromoindoles, bromotryptophans, and synthetic tripeptides.^23^ However, under the optimized reaction conditions reported therein for simple aryl bromides, no product formation was observed for the brominated peptidic substrates developed in this study (Table S3, entry 1). Using phenylacetylene (**1**) as the model alkyne, we were also unsuccessful in achieving product formation by changing the temperature alone with higher temperatures effecting substrate degradation (Table S3, entry 2). Thus, a comprehensive evaluation of reaction conditions was undertaken to reveal that at an intermediate temperature of 65 °C, a higher catalyst and the ligand loading were necessary for the Sonogashira coupling to be affected upon the longer, ribosomally derived peptide (Figure 3A, Table S3, entry 3). The observation that higher ligand and catalyst loading were necessary for the derivatization of peptides and proteins is not without precedent.^17^ We noted that increasing the temperature from 65 to 80 °C (Table S3, entry 5) led to abolition of product formation likely due to substrate degradation, suggesting that 65 °C is the optimal temperature for this reaction.

**Figure 3:**
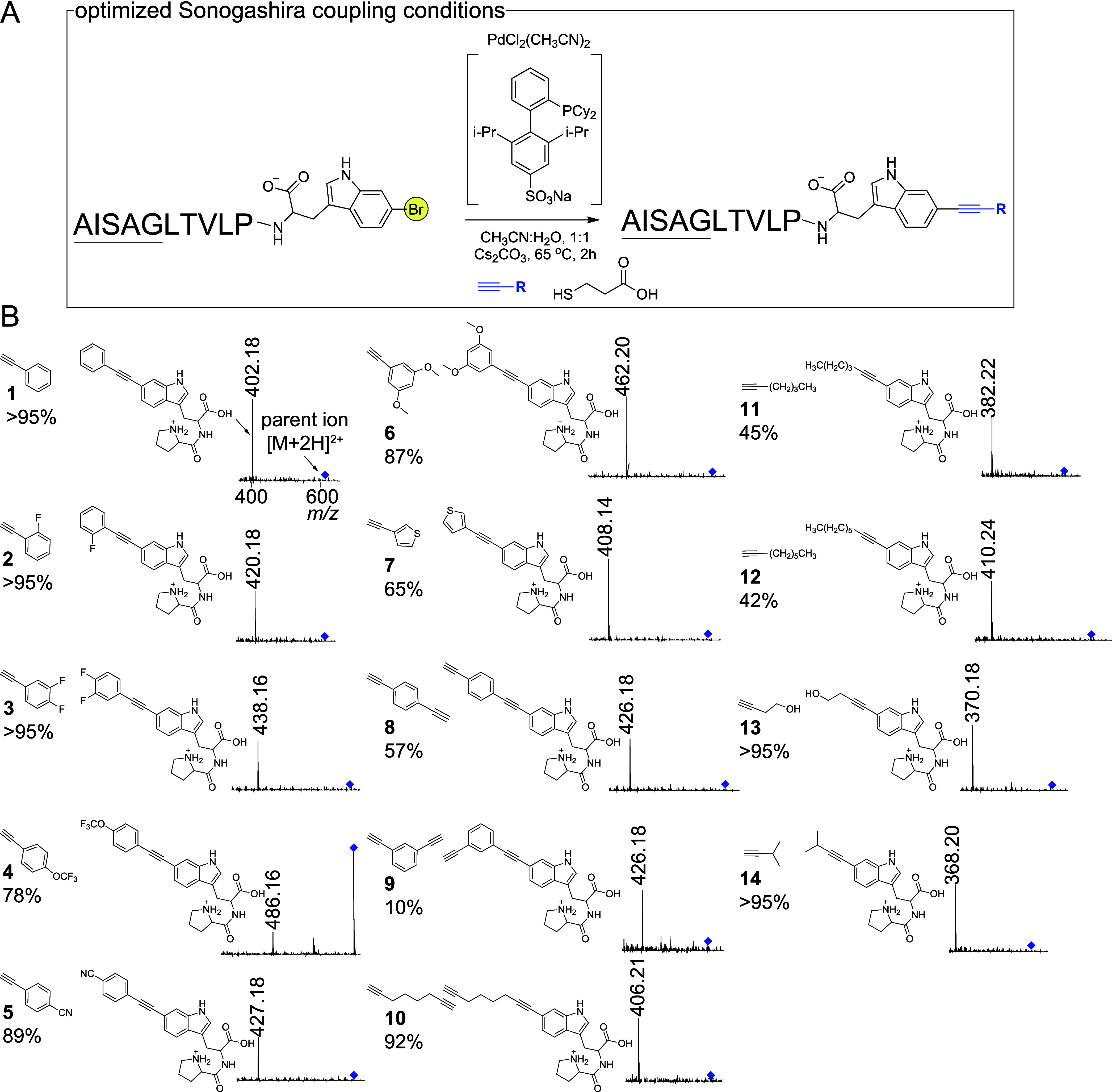
(A) Optimized Sonogashira reaction scheme with reaction conditions as follows: 1 mg of Br-peptide (1 equiv), 10 eq. terminal alkyne, 6.2 eq. Cs_2_CO_3_, degassed water (0.1 mL), MeCN (0.1 mL), [PdCl_2_(CH_3_CN)_2_] (15 mol %), ligand sXPhos (18 mol %), 65 °C, 2 h; all solvents purged with argon. Reactions were quenched by the addition of mercaptopropionic acid. (B) Yields for Sonogashira coupling of alkynes **1**–**14** with the SrpI-brominated peptide. Also illustrated are MS^2^ fragmentation spectra for each coupling product, wherein the diagnostic prolyl-(modified)Trp ion can be visualized. The [M+2H]^2+^ parent ions are illustrated by blue diamonds.

In line with Davis’ finding, scavenging the palladium by the addition of mercaptopropionic acid facilitated reaction monitoring by LC/MS.^24^ Using these optimized conditions, product yields using a panel of alkynes **1**–**14** were evaluated to establish the broad substrate scope of the Sonogashira coupling reaction conditions developed in this study. As before, reaction products were characterized using the Pro-(modified)Trp MS^2^ fragmentation ions (Figures 3B and S17–S44, Table S4).

Excellent product yields for a majority of aryl alkynes were observed. Using the cross-coupling strategy, we were able to introduce fluoro, cyano, and thiophene handles onto the ribosomal peptide, in addition to simple arenes with moderate to excellent yields (Figure 3B). In general, electron withdrawing groups on the aryl rings were associated with higher yields, likely due to higher acidity of the terminal alkyne hydrogen. Yields for coupling of long chain and branched aliphatic alkynes **11** and **12** were perhaps challenged by substrate solubility; the cross-coupling reactions with **13** and **14** proceeded with high yields.

In addition to querying the reaction scope, we explored whether aliphatic and aryl diynes could be coupled to the brominated peptide, with the motivation that the other unreacted alkyne terminus could serve as an additional reactive handle for a second round of bioorthogonal peptide derivatization. Moderate to excellent yields for the coupling of 1,4-diethynylbenzene (**8**) and 1,7-octadiyne (**10**) were observed to produce terminal alkyne bearing peptides **15** and **16**, respectively. Steric complexity in coordinating with the Pd complex could have compromised the yield for 1,3-diethynylbenzene (**9**) (Figure 3B).

Pursuant to the above-mentioned motivation of exploring diyne substrates for the Sonogashira cross-coupling reaction, we investigated if simple aryl and aliphatic azides could be coupled to **15** and **16** using the traditional copper-catalyzed alkyne–azide click reaction (CuAAC) to yield triazole products. In the single-pot reaction scheme thus devised, the Sonogashira reaction was not quenched by the addition of mercaptopropionic acid. Rather, the copper salt and ligand THPTA were introduced at the end of the Sonogashira reaction period. The CuAAC coupling was allowed to proceed at room temperature, followed by quenching by mercaptopropionic acid and analysis by LC/MS. Gratifyingly, the appropriate triazole products were observed representing hypermodified ribosomal peptides in excellent yields (Figures 4B and S45–S50, Table S5).

**Figure 4:**
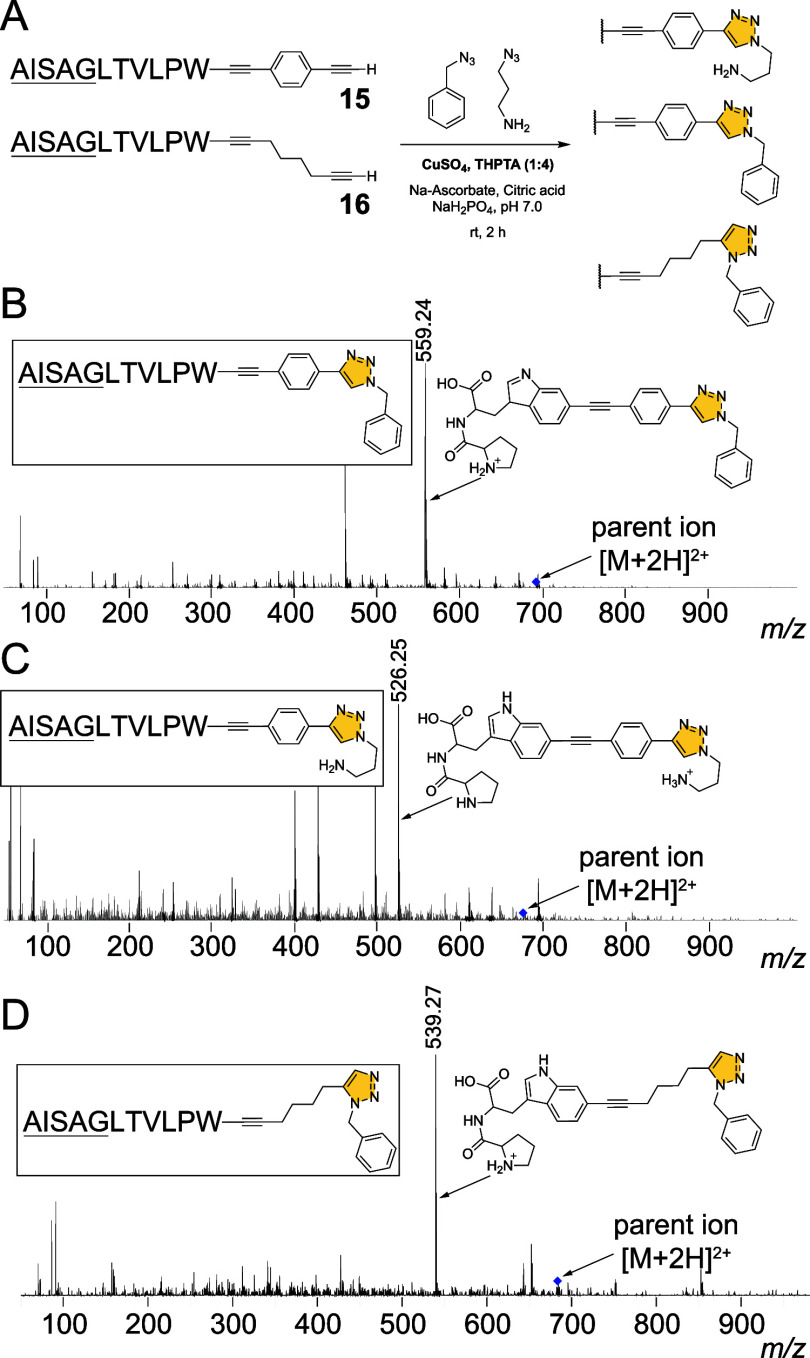
(A) Scheme for CuAAC coupling of azides to modified peptides **15** and **16**. (B) MS^2^ fragmentation spectra for the triazole products with characteristic MS^2^ fragmentation ions labeled. The [M+2H]^2+^ parent ions are illustrated by blue diamonds.

Taken together, these data establish the applicability of the RiPP halogenase SrpI and the Sonogashira reaction to extend the chemistry of ribosomally derived peptides. A ribosomal peptide with multiple other Trp and Tyr residues is regiospecifically brominated in high yield by SrpI; no passivation by mutagenesis of these other Trp and Tyr residues is required. While well-developed derivatization reactions for Cys, Lys, Tyr, and His side chains are now available, strategies for late-stage derivatization of the Trp side chain, particularly at the indole-4–7 positions, are rarified.^25−28^ Biocatalytic reactions can fill this void, particularly as applications are extended to long peptides and proteins that have multiple reactive handles and require mild and aqueous reaction conditions. At present, the reaction conditions herein perhaps limit applications to small peptides and thermostable proteins. Regardless, indole derivatization is exceptionally well represented in natural products and in medicinal chemistry.^29^ It is thus noteworthy that late-stage peptidic Trp-modifying enzymes are usually derived from natural product biosynthetic gene clusters exemplifying the applicability of natural product biosynthetic enzymes in biocatalytic applications.^30^

## Supporting Information Available

Comprehensive description of materials and methods used in this study, synthetic schemes, compound characterization data, and descriptions of enzyme reaction outcomes (PDF)

## References

[ref1] Young D. D.; Schultz P. G. Playing with the Molecules of Life. ACS Chem. Biol. 2018, 13, 854–870. 10.1021/acschembio.7b00974. 29345901 PMC6061972

[ref2] Noisier A. F. M.; Brimble M. A. C–H Functionalization in the Synthesis of Amino Acids and Peptides. Chem. Rev. 2014, 114, 8775–8806. 10.1021/cr500200x. 25144592

[ref3] Zhang C.; Vinogradova E. V.; Spokoyny A. M.; Buchwald S. L.; Pentelute B. L. Arylation Chemistry for Bioconjugation. Angew. Chem., Int. Ed. 2019, 58, 4810–4839. 10.1002/anie.201806009. PMC6433541 30399206

[ref4] Spicer C. D.; Triemer T.; Davis B. G. Palladium-Mediated Cell-Surface Labeling. J. Am. Chem. Soc. 2012, 134, 800–803. 10.1021/ja209352s. 22175226

[ref5] Chalker J. M.; Wood C. S. C.; Davis B. G. A Convenient Catalyst for Aqueous and Protein Suzuki–Miyaura Cross-Coupling. J. Am. Chem. Soc. 2009, 131, 16346–16347. 10.1021/ja907150m. 19852502

[ref6] Lee T.-K.; Manandhar B.; Kassees K. J.; Ahn J.-M. Peptide Ligation via the Suzuki–Miyaura Cross-Coupling Reaction. J. Org. Chem. 2020, 85, 1376–1384. 10.1021/acs.joc.9b02313. 31773962

[ref7] Montalbán-López M.; Scott T. A.; Ramesh S.; Rahman I. R.; van Heel A. J.; Viel J. H.; Bandarian V.; Dittmann E.; Genilloud O.; Goto Y.; Grande Burgos M. J.; Hill C.; Kim S.; Koehnke J.; Latham J. A.; Link A. J.; Martínez B.; Nair S. K.; Nicolet Y.; Rebuffat S.; Sahl H.-G.; Sareen D.; Schmidt E. W.; Schmitt L.; Severinov K.; Süssmuth R. D.; Truman A. W.; Wang H.; Weng J.-K.; van Wezel G. P.; Zhang Q.; Zhong J.; Piel J.; Mitchell D. A.; Kuipers O. P.; van der Donk W. A. New developments in RiPP discovery, enzymology and engineering. Nat. Prod. Rep. 2021, 38, 130–239. 10.1039/D0NP00027B. 32935693 PMC7864896

[ref8] Ortega M. A.; Cogan D. P.; Mukherjee S.; Garg N.; Li B.; Thibodeaux G. N.; Maffioli S. I.; Donadio S.; Sosio M.; Escano J.; Smith L.; Nair S. K.; van der Donk W. A. Two Flavoenzymes Catalyze the Post-Translational Generation of 5-Chlorotryptophan and 2-Aminovinyl-Cysteine during NAI-107 Biosynthesis. ACS Chem. Biol. 2017, 12, 548–557. 10.1021/acschembio.6b01031. 28032983 PMC5315687

[ref9] Harris L. A.; Saad H.; Shelton K. E.; Zhu L.; Guo X.; Mitchell D. A. Tryptophan-centric Bioinformatics Identifies new Lasso Peptide Modifications. Biochemistry 2024, 63, 865–879. 10.1021/acs.biochem.4c00035. 38498885 PMC11197979

[ref10] Montua N.; Thye P.; Hartwig P.; Kühle M.; Sewald N. Enzymatic Peptide and Protein Bromination: the BromoTrp tag. Angew. Chem., Int. Ed. 2024, 63, e202314961 10.1002/anie.202314961. 38009455

[ref11] Nguyen N. A.; Lin Z.; Mohanty I.; Garg N.; Schmidt E. W.; Agarwal V. An Obligate Peptidyl Brominase Underlies the Discovery of Highly Distributed Biosynthetic Gene Clusters in Marine Sponge Microbiomes. J. Am. Chem. Soc. 2021, 143, 10221–10231. 10.1021/jacs.1c03474. 34213321 PMC8780398

[ref12] Nguyen N. A.; Vidya F. N. U.; Yennawar N. H.; Wu H.; McShan A. C.; et al. Disordered regions in proteusin peptides guide post-translational modification by a flavin-dependent RiPP brominase. Nat. Commun. 2024, 15, 1265 10.1038/s41467-024-45593-5. 38341413 PMC10858898

[ref13] Chinchilla R.; Nájera C. The Sonogashira Reaction: A Booming Methodology in Synthetic Organic Chemistry. Chem. Rev. 2007, 107, 874–922. 10.1021/cr050992x. 17305399

[ref14] Krapf P.; Richarz R.; Urusova E. A.; Neumaier B.; Zlatopolskiy B. D. Seyferth–Gilbert Homologation as a Route to 18F-Labeled Building Blocks: Preparation of Radiofluorinated Phenylacetylenes and Their Application in PET Chemistry. Eur. J. Org. Chem. 2016, 2016 (3), 430–433. 10.1002/ejoc.201501377.

[ref15] Corona C.; Bryant B. K.; Arterburn J. B. Synthesis of a Biotin-Derived Alkyne for Pd-Catalyzed Coupling Reactions. Org. Lett. 2006, 8, 1883–1886. 10.1021/ol060458r. 16623575 PMC2523258

[ref16] Li J.; Lin S.; Wang J.; Jia S.; Yang M.; Hao Z.; Zhang X.; Chen P. R. Ligand-Free Palladium-Mediated Site-Specific Protein Labeling Inside Gram-Negative Bacterial Pathogens. J. Am. Chem. Soc. 2013, 135, 7330–7338. 10.1021/ja402424j. 23641876

[ref17] Li N.; Lim R. K. V.; Edwardraja S.; Lin Q. Copper-Free Sonogashira Cross-Coupling for Functionalization of Alkyne-Encoded Proteins in Aqueous Medium and in Bacterial Cells. J. Am. Chem. Soc. 2011, 133, 15316–15319. 10.1021/ja2066913. 21899368 PMC3184007

[ref18] Feuerstein M.; Doucet H.; Santelli M. Sonogashira cross-coupling reactions of aryl chlorides with alkynes catalysed by a tetraphosphine–palladium catalyst. Tetrahedron Lett. 2004, 45, 8443–8446. 10.1016/j.tetlet.2004.09.092.

[ref19] Hu H.; Yang F.; Wu Y. Palladacycle-Catalyzed Deacetonative Sonogashira Coupling of Aryl Propargyl Alcohols with Aryl Chlorides. J. Org. Chem. 2013, 78, 10506–10511. 10.1021/jo4014657. 24070411

[ref20] Nguyen N. A.; Agarwal V. A Leader-Guided Substrate Tolerant RiPP Brominase Allows Suzuki–Miyaura Cross-Coupling Reactions for Peptides and Proteins. Biochemistry 2023, 62, 1838–1843. 10.1021/acs.biochem.3c00222. 37272553 PMC10286304

[ref21] Mohanty I.; Nguyen N. A.; Moore S. G.; Biggs J. S.; Gaul D. A.; Garg N.; Agarwal V. Enzymatic Synthesis Assisted Discovery of Proline-Rich Macrocyclic Peptides in Marine Sponges. ChemBioChem. 2021, 22, 2614–2618. 10.1002/cbic.202100275. 34185944 PMC8415105

[ref22] Anderson K. W.; Buchwald S. L. General Catalysts for the Suzuki–Miyaura and Sonogashira Coupling Reactions of Aryl Chlorides and for the Coupling of Challenging Substrate Combinations in Water. Angew. Chem., Int. Ed. 2005, 44, 6173–6177. 10.1002/anie.200502017. 16097019

[ref23] Corr M. J.; Sharma S. V.; Pubill-Ulldemolins C.; Bown R. T.; Poirot P.; Smith D. R. M.; Cartmell C.; Abou Fayad A.; Goss R. J. M. Sonogashira diversification of unprotected halotryptophans, halotryptophan containing tripeptides; and generation of a new to nature bromo-natural product and its diversification in water. Chem. Sci. 2017, 8, 2039–2046. 10.1039/C6SC04423A. 28451322 PMC5398305

[ref24] Spicer C. D.; Davis B. G. Palladium-mediated site-selective Suzuki–Miyaura protein modification at genetically encoded aryl halides. Chem. Commun. 2011, 47, 1698–1700. 10.1039/c0cc04970k. 21206952

[ref25] Xiao Y.; Zhou H.; Shi P.; Zhao X.; Liu H.; Li X. Clickable tryptophan modification for late-stage diversification of native peptides. Sci. Adv. 2024, 10, eadp9958 10.1126/sciadv.adp9958. 38985871 PMC11235173

[ref26] Wang P.; Liu J.; Zhu X.; Kenry; Yan Z.; Yan J.; Jiang J.; Fu M.; Ge J.; Zhu Q.; Zheng Y. Modular synthesis of clickable peptides via late-stage maleimidation on C(7)-H tryptophan. Nat. Commun. 2023, 14, 3973 10.1038/s41467-023-39703-y. 37407547 PMC10322970

[ref27] Lee J. C.; Cuthbertson J. D.; Mitchell N. J. Chemoselective Late-Stage Functionalization of Peptides via Photocatalytic C2-Alkylation of Tryptophan. Org. Lett. 2023, 25, 5459–5464. 10.1021/acs.orglett.3c01795. 37462428 PMC10391624

[ref28] Kaplaneris N.; Puet A.; Kallert F.; Pöhlmann J.; Ackermann L. Late-stage C–H Functionalization of Tryptophan-Containing Peptides with Thianthrenium Salts: Conjugation and Ligation. Angew. Chem., Int. Ed. 2023, 62, e202216661 10.1002/anie.202216661. 36581584

[ref29] Duan S.-F.; Song L.; Guo H.-Y.; Deng H.; Huang X.; Shen Q.-K.; Quan Z.-S.; Yin X.-M. Research status of indole-modified natural products. RSC med. chem. 2023, 14, 2535–2563. 10.1039/D3MD00560G. 38107170 PMC10718587

[ref30] Kissman E. N.; Sosa M. B.; Millar D. C.; Koleski E. J.; Thevasundaram K.; Chang M. C. Y. Expanding chemistry through in vitro and in vivo biocatalysis. Nature 2024, 631, 37–48. 10.1038/s41586-024-07506-w. 38961155

