# Supplementary material for: Halogenase-Assisted Alkyne/Aryl Bromide Sonogashira Coupling for Ribosomally Synthesized Peptides
Source: J Am Chem Soc. 2024 Oct 23;146(44):30009–13. doi: 10.1021/jacs.4c12210 (PMC11544707; doi:10.1021/jacs.4c12210)
Supplement: Supplementary file 1 — ja4c12210_si_001.pdf [file ja4c12210_si_001.pdf]

SUPPLEMENTARY INFORMATION FOR:

Halogenase-assisted alkyne/aryl bromide Sonogashira coupling for ribosomally synthesized peptides

Nirmal Saha,[1] FNU Vidya,[1] Ramon Xie,[1] Vinayak Agarwal[1,2,]*

[1]School of Chemistry and Biochemistry, Georgia Institute of Technology, Atlanta, GA 30332, USA

[2]School of Biological Sciences, Georgia Institute of Technology, Atlanta, GA 30332, USA

*correspondence: vagarwal@gatech.edu, Ph: 404-385-3798

Supplementary Information document contains:

Supplementary Materials and Methods

Supplementary Tables S1–S5

Supplementary Figures S1–S50

Supplementary References

Supplementary Materials and Methods

General materials and instrumentation

All chemicals, solvents, and media components were obtained from Sigma-Aldrich, Fisher Scientific, and VWR, and used without further purification. Phusion High-Fidelity DNA polymerase and Gibson Assembly Master Mix were purchased from New England Biolabs. PrimeSTAR DNA polymerase Master Mix was purchased from Takara Bio. Reactions were monitored by thin layer chromatography (TLC) carried out on Supelco silica gel (60 F254) glass plates visualized under ultraviolet (UV) illumination. Silica gel (SiliaFlash GE60, 60–200 μm) was used for flash chromatography. Nuclear magnetic resonance (NMR) spectra were recorded on Bruker Avance III HD 400 MHz and 500 MHz instruments and calibrated using residual undeuterated solvent as the internal reference ($CDCl_3$ δ_H 7.26 and δ_C 77.16, MeOD δ_H 3.31 and δ_C 49.00, DMSO-d_6 δ_H 2.50 and δ_C 39.52). The splitting patterns were reported as s=singlet, d=doublet, t=triplet, q=quartet, m=multiplet, br=broad. Mass spectra were recorded on an Agilent 6530 LC/Q-ToF mass spectrometer with an electrospray ionization (ESI) source coupled to an Agilent 1260 ultra-high-performance liquid chromatography system equipped with a diode array detector.

Procedure for heterologous expression N-His$_6$-MBP-SrpE$^{(leader)}$-X$^{(core)}$ substrate peptides

Substrate peptides used in this study were fused to the SrpE leader sequence which was preceded by the maltose binding protein (MBP) and a N-terminal hexahistidine (N-His$_6$) tag to aid in peptide solubility and purification. The substrate peptides are henceforth designated as N-His$_6$-MBP-SrpE$^{(leader)}$-X$^{(core)}$ peptides with the X$^{(core)}$ being variable (Table S1).

For overexpression of N-His$_6$-MBP-SrpE$^{(leader)}$-X$^{(core)}$ substrate peptides, pET28(+) plasmid vector carrying the corresponding peptide coding sequence was transformed into *Escherichia coli* strain BL21(DE3). Colonies were grown under kanamycin antibiotic selection (50 μg/mL, final concentration) on Luria Bertani-agar (LB-agar) media for 16 h at 37 °C. A single colony was picked and inoculated in to 10 mL terrific broth (TB) supplemented with kanamycin for 16 h at 37 °C with shaking. The inoculum was used to initiate 1 L TB media supplemented with kanamycin. Cultures were incubated with shaking at 30 °C until the optical density measured at 600 nm wavelength (OD_{600}) reached 0.6. Cultures were cooled at 18 °C for 1 h before induction of protein expression by addition of 0.1 mM (final concentration) isopropyl-β-d-thiogalactopyranoside (IPTG). Cultures were further incubated at 18 °C with shaking for 24 h.

Purification protocol for N-His$_6$-MBP-SrpE$^{(leader)}$-X$^{(core)}$ substrate peptides

Ni-NTA affinity gel chromatography

E. coli cultures were harvested by centrifugation (3,000×g, 25 min, 4 °C) and resuspended in 50 mL lysis buffer (20 mM Tris-Cl (pH 7.9), 500 mM NaCl) and lysed by homogenization. The lysate was clarified by centrifugation at 36,000×g for 45 min at 4 °C. The supernatant was decanted and loaded on to a 5 mL His-Trap Ni-NTA column equilibrated with the lysis buffer at 4 °C. The column was washed with 30 mL wash buffer (20 mM Tris-Cl (pH 7.9), 500 mM NaCl, 30 mM imidazole). Bound proteins were then eluted using elution buffer B (20 mM Tris-Cl (pH 7.9), 500 mM NaCl, 250 mM imidazole) in five fractions of 8 mL volume each. Recombinant protein purity was analyzed by denaturing gel electrophoresis and the fractions containing protein of interest were pooled and dialyzed overnight against the buffer 20 mM Tris-Cl (pH 8.9), 50 mM KCl.

IEX chromatography

The dialyzed protein was loaded on to a 5 mL Hi-Trap IEX column equilibrated with the binding buffer (20 mM Tris-Cl (pH 8.9), 50 mM KCl). The column was washed with 30 mL wash buffer (20 mM Tris-Cl (pH 8.9), 100 mM KCl). Bound proteins were then eluted using elution buffer B (20 mM Tris-Cl (pH 8.9), 1 M KCl) in four fractions of 6 mL volume each. The purity of eluent fractions was analyzed by denaturing gel electrophoresis and pure fractions pooled. Protein concentration was measured by Bradford assay. Aliquots were frozen and stored at −80 °C.

Flavin reductase RebF and phosphite dehydrogenase PTDH were purified per protocols reported previously.[1]

Procedure for heterologous expression N-His$_6$-SrpI

For overexpression of N-His$_6$-SrpI, pET28(+) vector carrying corresponding coding sequence was co-transformed with plasmid pGro7 (Takara Biosciences) into *E. coli* BL21(DE3). Colonies were grown under antibiotic kanamycin (50 μg/mL, final concentration) and chloramphenicol (34 μg/mL, final concentration) on LB-agar media for 16 h. A single colony was picked and inoculated in 10 mL of TB media supplemented with kanamycin and chloramphenicol for 16 h at 37 °C. This inoculum was used to initiate 1 L TB media supplemented with kanamycin and chloramphenicol. Cultures were incubated with shaking at 30 °C until the OD_{600} reached 0.6. Cultures were cooled at 18 °C for 1 h before induction of protein expression by addition of 0.3 mM (final concentration) IPTG and 250 mg L-arabinose. The cultures were allowed to further incubate for 48 h at 18 °C with shaking.

Purification protocol for N-His$_6$-SrpI

Ni-NTA affinity gel chromatography

E. coli cultures were harvested by centrifugation as above, and cell pellets were resuspended in lysis buffer A (20 mM Tris-Cl (pH 7.9), 500 mM NaCl). Cells were lysed by homogenization. The lysate was clarified by centrifugation at 36,000×g for 60 min at 4 °C. The supernatant was loaded on to a 5 mL His-Trap Ni-NTA column at 4 °C. The column was washed extensively with wash buffer B (20 mM Tris-Cl (pH 7.9), 500 mM NaCl, 30 mM imidazole), and protein was eluted using a linear gradient from 0% to 100% elution buffer B (20 mM Tris-Cl (pH 7.9), 500 mM NaCl, 250 mM imidazole). Protein purity was analyzed by denaturing gel electrophoresis (gel image, right), and fractions containing protein of interest were pooled and dialysis overnight in the buffer containing 20 mM Tris-Cl (pH 8.9), 50 mM KCl.

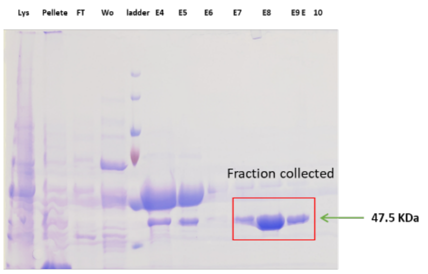

SDS-PAGE 12%

IEX chromatography

After overnight dialysis, the protein was loaded on to a 5 mL Hi-Trap IEX column equilibrated with the binding buffer (20 mM Tris-Cl (pH 8.9), 50 mM KCl) at 4 °C. Bound proteins were eluted using elution buffer (20 mM Tris-Cl (pH 8.9), 1 M KCl) in four fractions of 6 mL volume each. The purity of eluent fractions was checked by denaturing gel electrophoresis and pure fractions pooled. Protein concentration was measured by Bradford assay.

SrpI-catalyzed bromination of N-His$_6$-MBP-SrpE$^{(leader)}$-X$^{(core)}$ substrates

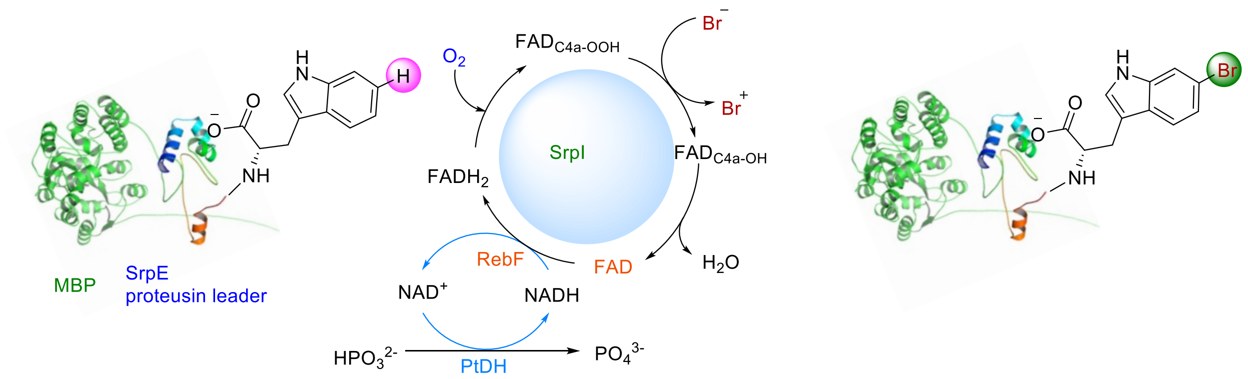

Analytical scale bromination reactions were performed in 200 µL volume containing 50 mM HEPES-Na (pH 7.9), 20 mM KBr, 25 µM FAD, 0.5 mM NAD$^+$, 5 mM Na_2HPO_3, 2.5 µM flavin reductase (RebF), 2.5 µM PTDH, 25 µM substrate peptide, 5 µM SrpI, and 0.01 µg/µL catalase. After 20 h incubation at 30 °C,

reactions were quenched by addition of the protease Glu-C. After overnight incubation at room temperature, the reactions treated with Glu-C were desalted and analyzed by LC/MS.

SrpI-catalyzed preparative scale bromination of N-His$_6$-MBP-SrpE$^{(leader)}$-LTVLPW$^{(core)}$

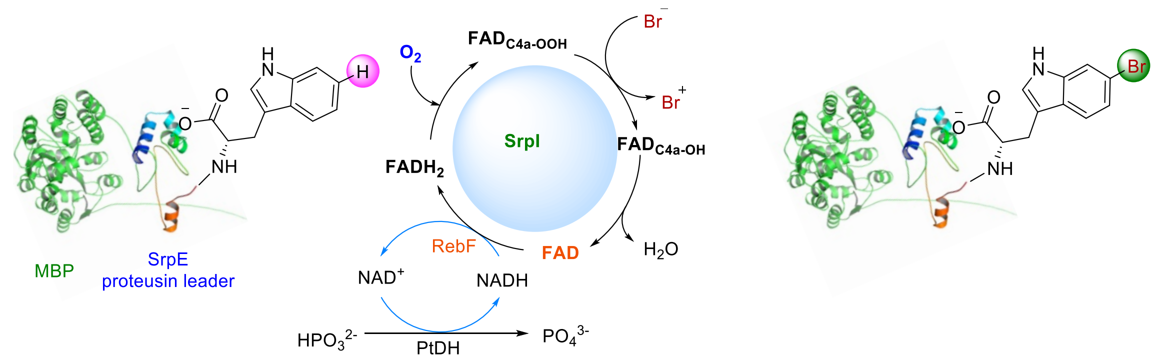

Component	Stock Conc.	Final Conc.	Volume (μL)
HEPES-Na (pH 7.9)	1 M	50 mM	500
H_2O			5,640.4
KBr	2.5 M	20 mM	80
FAD	5 mM	25 μM	50
NAD^+	125 mM	0.625 mM	50
Na_2HPO_3	1.25 M	6.25 mM	50
PTDH	310 μM	2.5 μM	80.6
SrpI	40 μM	10 μM	2,500
N-His$_6$-MBP-SrpE$^{(leader)}$-LTVLPW$^{(core)}$	600 μM	50 μM	833.3
Catalase	10 ng/μL	0.05 ng/μL	50
RebF	150 μM	2.5 μM	166
Total volume			10,000
30 °C, 20 h			

Per the table above, for a 10 mL preparative scale reaction in a 50 mL conical tube were added 50 mM HEPES-Na (pH 7.9), 20 mM KBr, 25 μM FAD, 0.5 mM NAD^+, 5 mM Na_2HPO_3, 2.5 μM PTDH, 5 μM SrpI, 25 μM substrate peptide, 0.01 μg/μL catalase and 2.5 μM RebF. The reaction mixture was mixed by tapping and incubated at 30 °C for 20 h. The reaction was then desalted into water using PD-10 desalting columns operating under gravity. Next, 12 mL of the desalted reaction mixture was digested with Glu-C (50 μL,10 ng/μL stock concentration) overnight at room temperature followed by incubation with 1 mL Ni-NTA affinity beads for 30 min with shaking. The beads were recovered by centrifugation at 2,000×g for 15 min and discarded. To the supernatant was added an equal volume of MeCN and the solution was vigorously vortexed. Precipitated proteins were removed by centrifugation at 36,000×g for 45 min and the supernatant

decanted into a round bottom flask. The MeCN was removed using a rotary evaporator. The resultant liquid was frozen and lyophilized to yield 10 mg of a white-fluffy brominated peptide product. The product identity was verified using LC/MS.

Synthesis of sXPhos

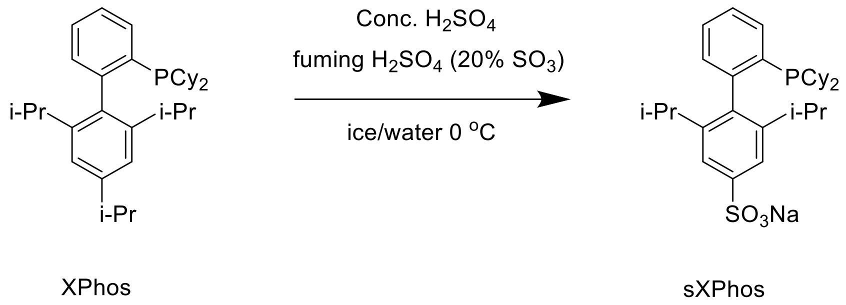

In a 25 mL round bottom flask (oven dried and fitted with a Teflon-coated magnetic stir bar and rubber septum), dicyclohexyl-(2',4',6'-triisopropyl-biphenyl-2-yl)-phosphane (476 mg, 1.00 mmol) was combined with CH_2Cl_2 (1.0 mL). The resulting solution was cooled to 0 °C using an ice/water bath before concentrated H_2SO_4 (1.0 mL) and fuming sulfuric acid (3.0 mL, 20% SO_3) were added dropwise. The mixture was allowed to reach room temperature and stirred for 24 h. It was then cooled back to 0 °C using an ice/water bath, and 10 g of crushed ice was introduced. The solution turned cloudy and white. Then, the solution was neutralized by dropwise addition of 6 M aqueous NaOH (~40.0 mL; pH ~7.0 as indicated by pH paper). The resulting aqueous phase was extracted with CH_2Cl_2 (3×50 mL) and concentrated under reduced pressure yielding a beige solid. The crude product was dissolved in a minimal amount of cold methanol (~20 mL), filtered, and the solvent was removed using a rotary evaporator. This process was repeated, ultimately furnishing the desired product sXPhos as a beige solid (180 mg, 34% yield).

^{1}H NMR (400 MHz, CD_3OD) δ: 7.52 (s, 2H), 7.46 (m, 1H), 7.22 (m, 2H), 6.90 (m, 1H), 2.24 (m, 1H), 0.71-1.83 (34H).

^{31}P NMR (162 MHz, CD_3OD) δ: −11.47.

Spectroscopic data are in excellent agreement with those reported by Buchwald and co-workers.[2] Exact Mass: $[M+H]^+$ 515.2743, Observed Mass: $[M+H]^+$ 515.2783.

Procedure for Sonogashira coupling of alkynes with brominated peptide

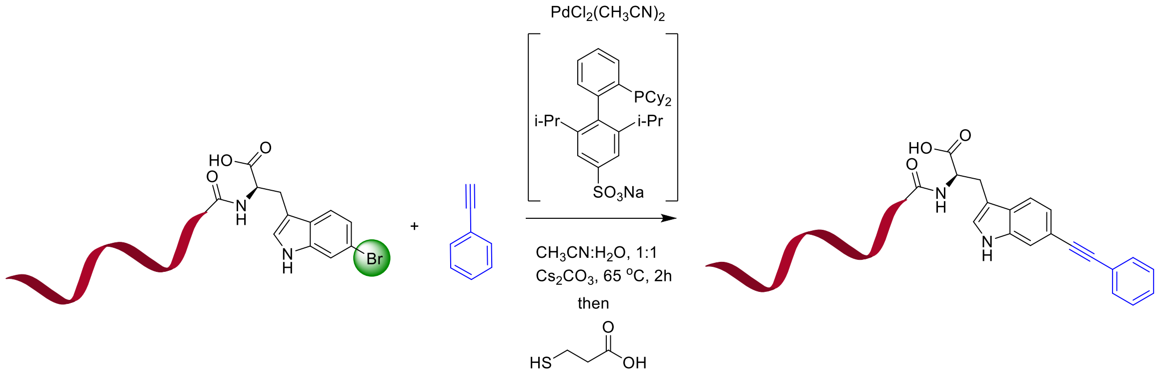

A two-neck sealed tube with a stir bar was charged with the brominated peptide (2.0 mg, 1.6 μmol, 1.0 eq), sXPhos (0.3 mg, 0.6 μmol, 0.13 eq, 18 mol%) and $CsCO_3$ (3.5 mg, 10 μmol, 6.2 eq). The tube was sealed and flushed with argon. A stock solution of *bis*(acetonitrile)dichloropalladium(II) (0.1 mg, 0.5 μmol, 0.12 eq, 15 mol%) in degassed CH_3CN:H_2O (1:1, 0.2 mL) was prepared. The catalyst stock solution was injected into the sealed tube. The alkyne substrate (16 μmol, 10.0 eq) was then injected into the tube. The reaction mixture was stirred and then heated at 65 °C for 2 h in an oil bath. The reaction mixture was cooled to room temperature and transferred to a microcentrifuge tube and centrifuged at 16,000×g for 5 min. The supernatant was collected, and the reaction quenched by the addition of 3-mercaptopropionic acid (100 μL of 20 μL/mL stock solution in water). The reaction mixture was centrifuged again (16,000×g, 30 min) and the supernatant was analyzed by LC/MS. All reactions were performed in triplicate.

Procedure for LC/MS characterization of Sonogashira coupling reactions

Liquid chromatography (LC) was performed using an Agilent Poroshell 120 EC-C_{18} (2.7 μm, 4.6×100 mm) reverse phase column at a flow rate of 0.3 mL/min with binary solvent A: water (0.1% FA) and B: MeCN (0.1% FA). The following solvent time course was used following sample injection: 5% B for 5 min, linear gradient to 100% B over 18 min, 100% B 4 min, linear gradient to 5% B over 2 min, 5% B for 6 min. MS spectra were acquired in the positive ionization mode. MS^1 spectra were obtained at a range of *m/z* 400–2000. MS^2 spectra in the data-dependent acquisition mode were obtained at a range of *m/z* 50–3000. A calibration curve was constructed to correlate the MS abundance of the N-His$_6$-MBP-SrpE$^{(leader)}$-LTVLPW$^{(core)}$ substrate peptide, as detected using LC/MS, with protein

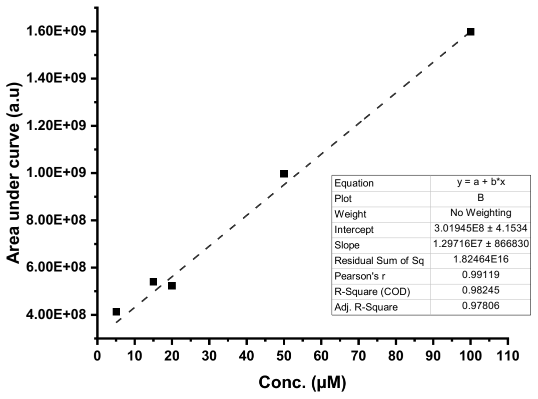

concentration. The area under the extracted ion chromatograms (EICs) for the $[M+2H]^{2+}$ ion corresponding to the Glu-C digested substrate peptide fragment H-AISAGLTVLPW-OH was plotted against protein concentration. It is important to note that throughout this study, a constant concentration of 25 µM substrate peptide was used in all enzymatic assays. Consequently, both the substrate and product peptide abundances were maintained at or below 25 µM ensuring they fell within the linear detection range of the mass spectrometer.

Procedure for alkyne/azide click chemistry

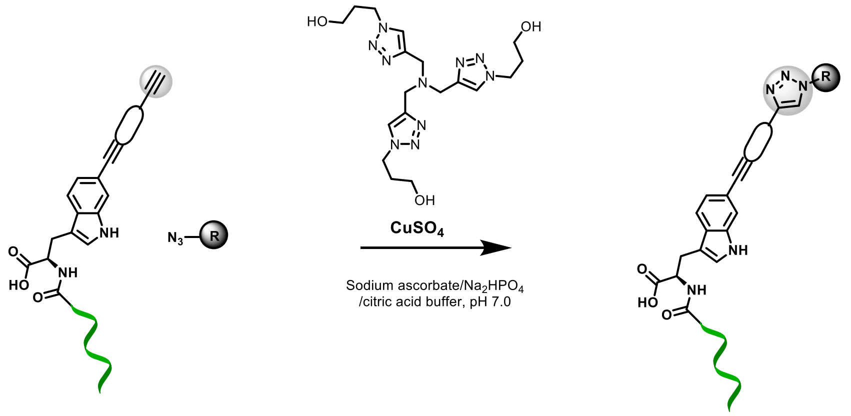

Azide solutions were prepared at a concentration of 100 µM. Sodium ascorbate/Na_2HPO_4/citric acid buffer was prepared as an aqueous neutral buffer (pH 7.0). This buffer was made by dissolving sodium ascorbate (0.248 g, 1.25 mmol), Na_2HPO_4 (0.7 g, 4.93 mmol), and citric acid (0.487 g, 2.54 mmol) in water, making a total volume of 10 mL. A solution of the peptide alkyne, as shown in the left side of the scheme above, was prepared at a concentration of 10 µM in MeCN. An aqueous solution containing $CuSO_4$ and THPTA was also prepared, with $CuSO_4$ at a 25 mM concentration and THPTA at a 100 mM concentration, by dissolving $CuSO_4$ (4.0 mg, 25 µmol) in 1 mL of water and THPTA (8.7 mg, 100 µmol) in another 1 mL of water. MeCN was used as the solvent.

After preparing all the reagents, they were combined in a 1.5 mL microcentrifuge tube as follows: freshly prepared $CuSO_4$ (5 µL) and ligand THPTA (5 µL) solutions were first mixed. Then, 40 µL of the sodium ascorbate/Na_2HPO_4/citric acid buffer solution was added. At this point, the tube was closed to limit exposure to oxygen. Subsequently, 40 µL of the alkyne stock solution and 10 µL of the azide solution were added sequentially into the tube. The tube was then closed again and mixed by inverting several times to ensure thorough mixing. The microcentrifuge tube was sealed and incubated at 30 °C for 2 h. After the incubation period, the reaction mixture was diluted with 100 µL of MeOH and 100 µL of MeCN. The mixture in each sample was then analyzed using HPLC-MS.

SUPPLEMENTARY TABLES

Supplementary Table S1: Substrate peptide sequences used in this study with core sequences in boldface

Name	Amino acid sequence
(N-His$_6$-MBP)	MGSSHHHHHH†SSGLVPRGS§HMKIEEGKLVIWINGDKGYNGLA<br>EVGKKFEKDTGIKVTVEHPDKLEEKFPQVAATGDGPDIIFWAH<br>DRFGGYAQSGLLAEITPDKAFQDKLYPFTWDAVRYNGKLIAYP<br>IAVEALSLIYNKDLLPNPPKTWEEIPALDKELKAKGKSALMFN<br>LQEPYFTWPLIAADGGYAFKYENGKYDIKDVGVDNAGAKAGLT<br>FLVDLIKNKHMNADTDYSIAEAAFNKGETAMTINGPWAWSNID<br>TSKVNYGVTVLPTFKGQPSKPFVGVLSAGINAASPNKELAKEF<br>LENYLLTDEGLEAVNKDKPLGAVALKSYEEELAKDPRIAATME<br>NAQKGEIMPNIPQMSAFWYAVRTAVINAASGRQTVDEALKDAQ<br>TNSSSHHHHHH†ANSVPLVPRGS§ENLYFQS‡GS
N-His$_6$-MBP-SrpE$^{(leader)}$-<br>**LPW**$^{(core)}$	(N-His$_6$-MBP)-MRSGDDMLQHLVEKSALDADFRQQLLADPKSTISQELGIS<br>IPESMTIRVHESDMETVHLALPPDPNLTEEQLEAISAG-**LPW**
N-His$_6$-MBP-SrpE$^{(leader)}$-<br>**LTVPW**$^{(core)}$	(N-His$_6$-MBP)-MRSGDDMLQHLVEKSALDADFRQQLLADPKSTISQELGIS<br>IPESMTIRVHESDMETVHLALPPDPNLTEEQLEAISAG-**LTVPW**
N-His$_6$-MBP-SrpE$^{(leader)}$-<br>**LTVLPW**$^{(core)}$	(N-His$_6$-MBP)-MRSGDDMLQHLVEKSALDADFRQQLLADPKSTISQELGIS<br>IPESMTIRVHESDMETVHLALPPDPNLTEEQLEAISAG-**LTVLPW**
N-His$_6$-MBP-SrpE$^{(leader)}$-<br>**LTVLVALPW**$^{(core)}$	(N-His$_6$-MBP)-MRSGDDMLQHLVEKSALDADFRQQLLADPKSTISQELGIS<br>IPESMTIRVHESDMETVHLALPPDPNLTEEQLEAISAG-**LTVLVALPW**
N-His$_6$-MBP-SrpE$^{(leader)}$-<br>**LTVLVPLLVPW**$^{(core)}$	(N-His$_6$-MBP)-MRSGDDMLQHLVEKSALDADFRQQLLADPKSTISQELGIS<br>IPESMTIRVHESDMETVHLALPPDPNLTEEQLEAISAG-**LTVLVPLLVPW**

†His$_6$ tag, § thrombin cleavage site, ‡TEV cleavage site

Supplementary Table S2: Calculated and observed masses of substrate peptides with different core sequences and corresponding brominated product peptides obtained after Glu-C digestion along with the product yields for reactions performed in triplicate

Substrates/Products	Calculated Mass	Observed Mass	Conversion (%)		
N-His$_6$-MBP- SrpE-LPW	814.4458 [M+H]$^+$<br>407.7265 [M+2H]$^{2+}$	814.4439 [M+H]$^+$<br>407.756 [M+2H]$^{2+}$			
N-His$_6$-MBP-SrpE-LPW-**Br**	892.3563 [M+H]$^+$<br>446.6818 [M+2H]$^{2+}$	892.3622 [M+H]$^+$<br>446.6847 [M+2H]$^{2+}$	9	20	12
N-His$_6$-MBP-SrpE-LTVPW	1014.5619 [M+H]$^+$<br>507.7846 [M+2H]$^{2+}$	1014.5661 [M+H]$^+$<br>507.7878 [M+2H]$^{2+}$			
N-His$_6$-MBP-SrpE -LTVPW-**Br**	1092.4724 [M+H]$^+$<br>546.7398 [M+2H]$^{2+}$	1092.4855 [M+H]$^+$<br>546.7490 [M+2H]$^{2+}$	91	87	84
N-His$_6$-MBP-SrpE-LTVLPW	1127.6459 [M+H]$^+$<br>564.3266 [M+2H]$^{2+}$	1127.6511 [M+H]$^+$<br>564.3325 [M+2H]^{2}			
N-His$_6$-MBP-SrpE-LTVLPW-**Br**	1205.5565 [M+H]$^+$<br>603.2819 [M+2H]$^{2+}$	1205.5746 [M+H]$^+$<br>603.2988 [M+2H]$^{2+}$	95	71	82
N-His$_6$-MBP-SrpE-LTVLVALPW	1410.8356 [M+H]$^+$<br>705.9214 [M+2H]$^{2+}$	1410.8454 [M+H]$^+$<br>705.9349 [M+2H]$^{2+}$			
N-His$_6$-MBP-SrpE-LTVLVALPW-**Br**	1488.7461 [M+H]$^+$<br>744.8767 [M+2H]$^{2+}$	1488.7502 [M+H]$^+$<br>744.8813 [M+2H]$^{2+}$	9	7	9
N-His$_6$-MBP-SrpE-LTVLVPLLVPW	1649.0037 [M+H]$^+$<br>825.0055 [M+2H]$^{2+}$	1649.0158 [M+H]$^+$<br>825.0303 [M+2H]$^{2+}$			
N-His$_6$-MBP-SrpE-LTVLVPLLVPW-**Br**	1726.9142 [M+H]$^+$<br>863.9607 [M+2H]$^{2+}$	1726.9230 [M+H]$^+$<br>863.9683 [M+2H]$^{2+}$	25	24	14

Supplementary Table S3: Conditions[a] for the Sonogashira cross-coupling reaction of Br-peptide substrate (AISAGLTVLPW-Br) with phenylacetylene

Entry	Substrate	Cat(mol%)	Ligand(mol%)	Alkyne(eq.)	Temp.	Time/h	Conv./%
1	AISAGLTVLPW-Br	5	15	3	100	18	N.D[d]
2	AISAGLTVLPW-Br	5	15	3	rt[e]	18	N.D
3	AISAGLTVLPW-Br	15	18	10	65	2	>95[b]
4	AISAGLTVLPW-Br	15	18	10	65	2	60[c]
5	AISAGLTVLPW-Br	10	15	3	80	18	N.D
6	AISAGLTVLPW-Br	10	15	3	65	18	62
7	AISAGLTVLPW-Br		15	10	65	2	N.D
8	AISAGLTVLPW-Br	15		10	65	2	N.D

[a]substrate peptide (1.6 µmol, 1.0 eq), *bis*$PdCl_2(CH_3CN)_2$ catalyst, sXPhos ligand, Phenylacetylene, $CsCO_3$ (6.2 eq), CH_3CN/H_2O (1:1, 0.2 mL), 2-neck sealed tube, temperature in °C, solvent and solid purged with argon

[b]yield determined by LC/MS

[c]yield determined by LC/MS without argon purging

[d]product not detected

[e]the Sonogashira reaction likely does not proceed at room temperature leading to no product formation

Supplementary Table S4: Calculated and observed masses of the substrate (AISAGLTVLPW-Br) and the Sonogashira cross-coupling product peptides in this study

No.	Substrates/Products	Calculated Mass	Observed Mass
	AISAGLTVLPW-Br	1205.5565 $[M+H]^+$<br>603.2819 $[M+2H]^{2+}$	1205.5746 $[M+H]^+$<br>603.2988 $[M+2H]^{2+}$
1	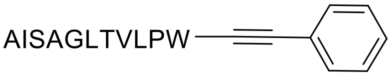	1227.6773 $[M+H]^+$<br>614.3423 $[M+2H]^{2+}$	1227.6742 $[M+H]^+$<br>614.3549 $[M+2H]^{2+}$
2	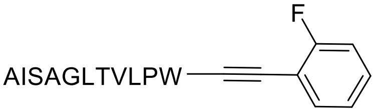	1245.6678 $[M+H]^+$<br>623.3376 $[M+2H]^{2+}$	1245.7061 $[M+H]^+$<br>623.3609 $[M+2H]^{2+}$
3	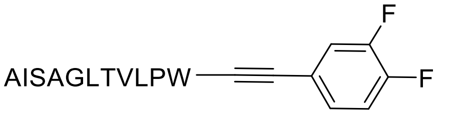	1263.6584 $[M+H]^+$<br>632.3329 $[M+2H]^{2+}$	1263.6584 $[M+H]^+$<br>632.3477 $[M+2H]^{2+}$
4	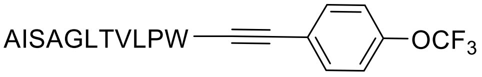	1311.6596 $[M+H]^+$<br>656.3334 $[M+2H]^{2+}$	1311.6668 $[M+H]^+$<br>656.3403 $[M+2H]^{2+}$
5	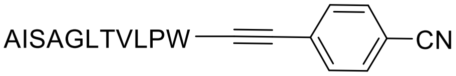	1252.6725 $[M+H]^+$<br>626.8366 $[M+2H]^{2+}$	1252.6785 $[M+H]^+$<br>626.8466 $[M+2H]^{2+}$
6	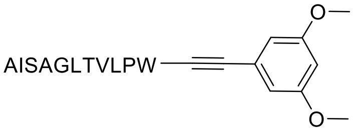	1287.6984 $[M+H]^+$<br>644.3528 $[M+2H]^{2+}$	1287.7140 $[M+H]^+$<br>644.3660$[M+2H]^{2+}$
7	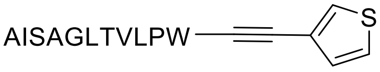	1233.6367 $[M+H]^+$<br>617.3271 $[M+2H]^{2+}$	1233.6384 $[M+H]^+$<br>617.3205 $[M+2H]^{2+}$
8	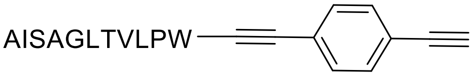	1251.6773 $[M+H]^+$<br>626.3423 $[M+2H]^{2+}$	1251.6871 $[M+H]^+$<br>626.3496 $[M+2H]^{2+}$
9	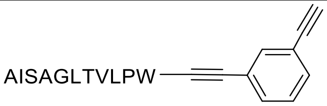	1251.6773 $[M+H]^+$<br>626.3423 $[M+2H]^{2+}$	1251.7251 $[M+H]^+$<br>626.3655 $[M+2H]^{2+}$
10	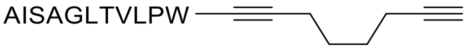	1231.7086 $[M+H]^+$<br>616.3579 $[M+2H]^{2+}$	1231.6937 $[M+H]^+$<br>616.3526 $[M+2H]^{2+}$
11	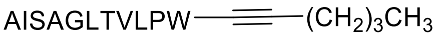	1207.7086 $[M+H]^+$<br>604.3579 $[M+2H]^{2+}$	1207.7505 $[M+H]^+$<br>617.3820 $[M+2H]^2$
12	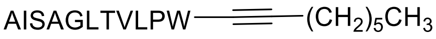	1235.7399 $[M+H]^+$<br>618.3817 $[M+2H]^{2+}$	1235.7487 $[M+H]^+$<br>618.3817 $[M+2H]^{2+}$
13	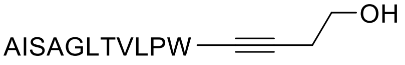	1195.6722 $[M+H]^+$<br>598.3397 $[M+2H]^{2+}$	1195.7087 $[M+H]^+$<br>598.3577 $[M+2H]^{2+}$
14	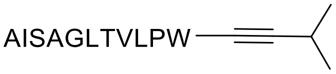	1193.6929 $[M+H]^+$<br>597.3501 $[M+2H]^{2+}$	1193.6996 $[M+H]^+$<br>597.3576 $[M+2H]^{2+}$

Supplementary Table S5: Calculated and observed masses of the substrate (AISAGLTVLPW-alkyne) and the CuAAC product peptides in this study.

	Substrates/Products	Calculated Mass (MS^2)	Observed Mass (MS^2)
**Substrate**	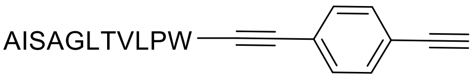	626.3423 $[M+2H]^{2+}$	626.3423 $[M+2H]^{2+}$
**product**	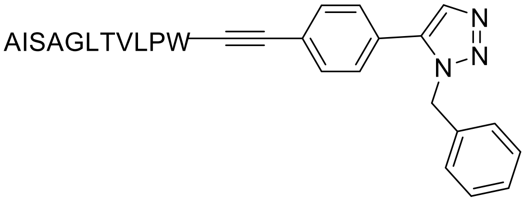	692.87 $[M+2H]^{2+}$	692.87 $[M+2H]^{2+}$
**product**	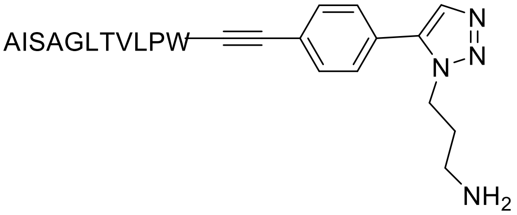	673.38 $[M+2H]^{2+}$	673.38 $[M+2H]^{2+}$
**Substrate**	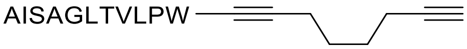	616.35 $[M+2H]^{2+}$	616.35 $[M+2H]^{2+}$
**product**	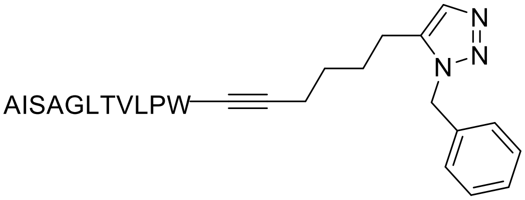	682.88 $[M+2H]^{2+}$	682.88 $[M+2H]^{2+}$

Supplementary Figures

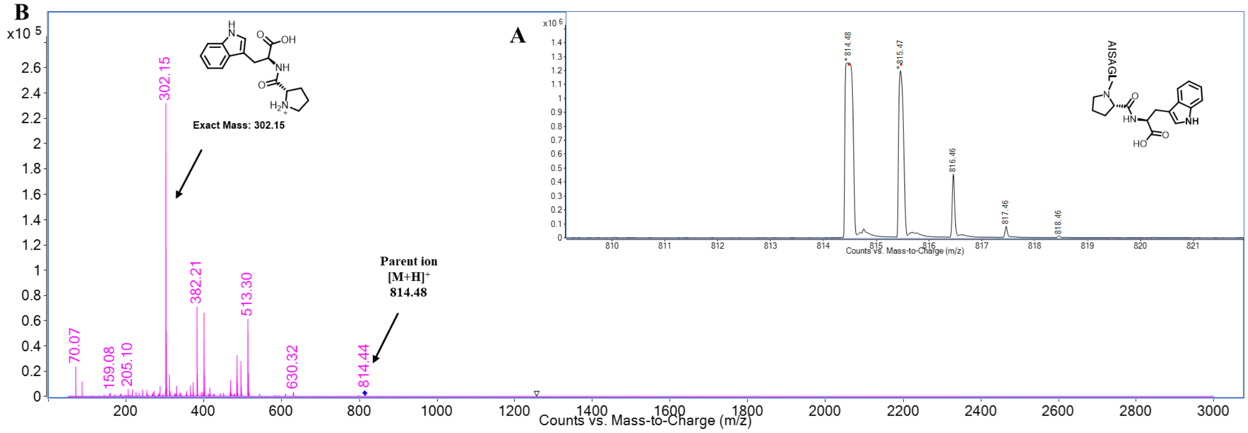

Figure S1: MS^1 and MS^2 spectra of the substrate peptide N-His$_6$-MBP-SrpE$^{(leader)}$-LPW$^{(core)}$ after digestion with Glu-C. (**A**) The isotopic distribution of $[M+2H]^{2+}$ ions corresponding to the Glu-C digestion product. (**B**) MS^2 fragmentation spectra for the Glu-C digested product. The Pro-Trp daughter ion is structurally annotated. The $[M+2H]^{2+}$ parent ion is labeled.

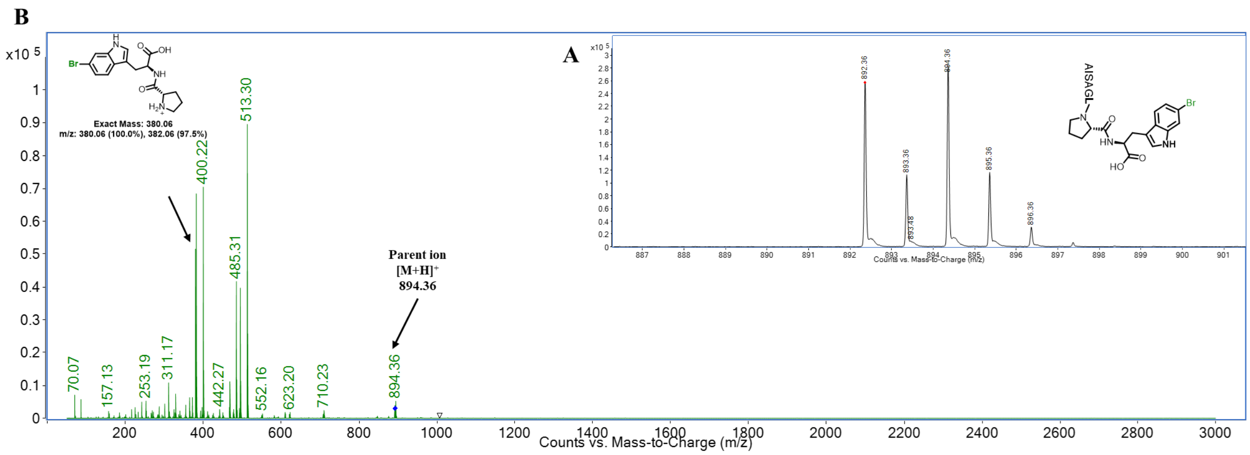

Figure S2: MS[1] and MS[2] spectra of brominated product N-His$_6$-MBP-SrpE$^{(leader)}$-LPW$^{(core)}$ after digestion with Glu-C. (**A**) The isotopic distribution of $[M+2H]^{2+}$ ions corresponding to the Glu-C digestion product. (**B**) MS[2] fragmentation spectra for the Glu-C digested product. The Pro-Trp daughter ion is structurally annotated and denotes that the bromination is localized to the C-terminal Trp residue. The $[M+2H]^{2+}$ parent ion is labeled.

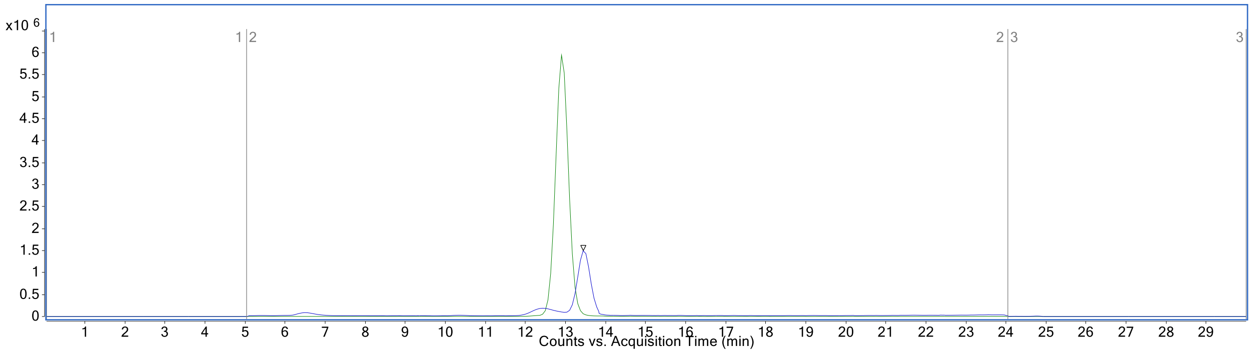

Figure S3: LC/MS analysis of the SrpI-catalyzed bromination of N-His$_6$-MBP-SrpE$^{(leader)}$-LPW$^{(core)}$ peptide after digestion with Glu-C. Extracted ion chromatograms (EICs) corresponding to the unmodified substrate and the brominated product are illustrated in green and blue, respectively. Area under these EICs was used to calculate the yield of the SrpI-catalyzed bromination reaction.

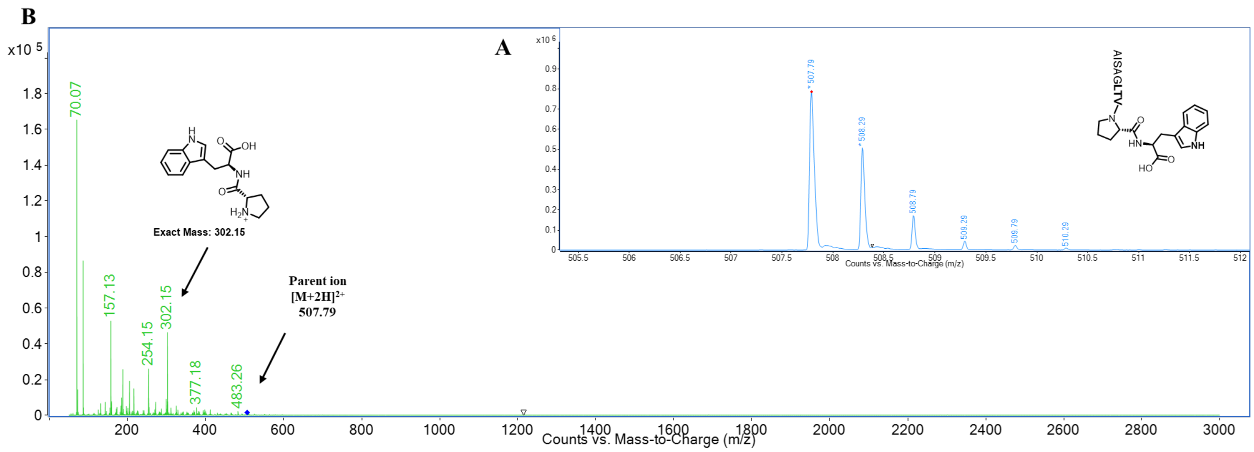

Figure S4: MS[1] and MS[2] spectra of the substrate peptide N-His$_6$-MBP-SrpE$^{(leader)}$-LTVPW$^{(core)}$ after digestion with Glu-C. (**A**) The isotopic distribution of $[M+2H]^{2+}$ ions corresponding to the Glu-C digestion product. (**B**) MS[2] fragmentation spectra for the Glu-C digested product. The Pro-Trp daughter ion is structurally annotated. The $[M+2H]^{2+}$ parent ion is labeled.

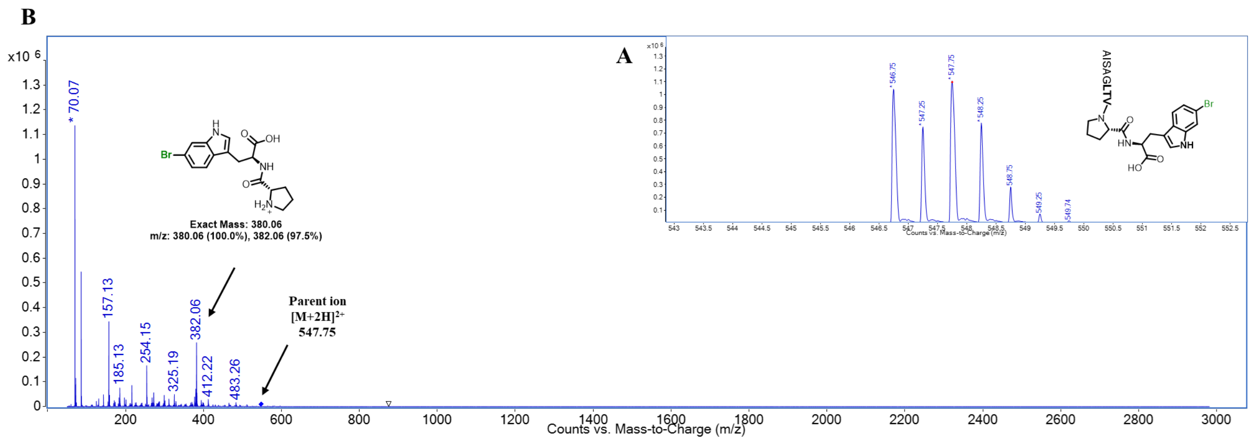

Figure S5: MS[1] and MS[2] spectra of brominated product N-His$_6$-MBP-SrpE$^{(leader)}$-LTVPW$^{(core)}$ after digestion with Glu-C. (**A**) The isotopic distribution of $[M+2H]^{2+}$ ions corresponding to the Glu-C digestion product. (**B**) MS[2] fragmentation spectra for the Glu-C digested product. The Pro-Trp daughter ion is structurally annotated and denotes that the bromination is localized to the C-terminal Trp residue. The $[M+2H]^{2+}$ parent ion is labeled.

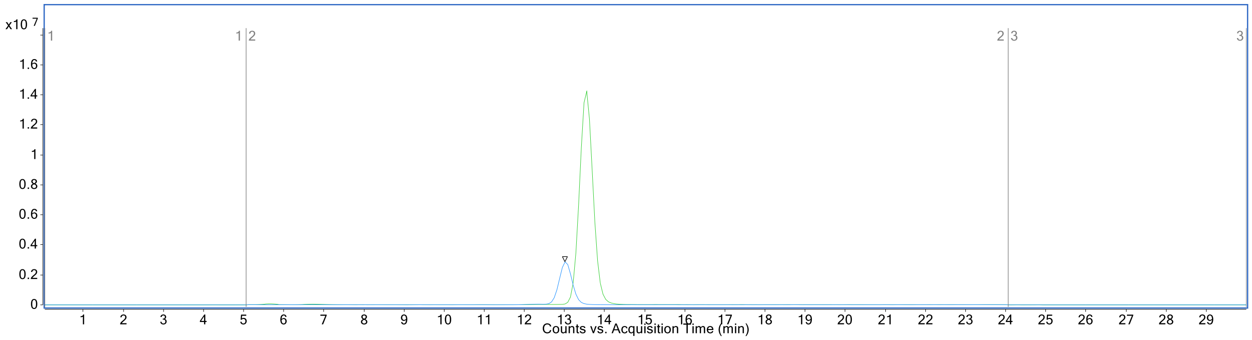

Figure S6: LC/MS analysis of the SrpI-catalyzed bromination of N-His$_6$-MBP-SrpE$^{(leader)}$-LTVPW$^{(core)}$ peptide after digestion with Glu-C. Extracted ion chromatograms (EICs) corresponding to the unmodified substrate and the brominated product are illustrated in blue and green, respectively. Area under these EICs was used to calculate the yield of the SrpI-catalyzed bromination reaction.

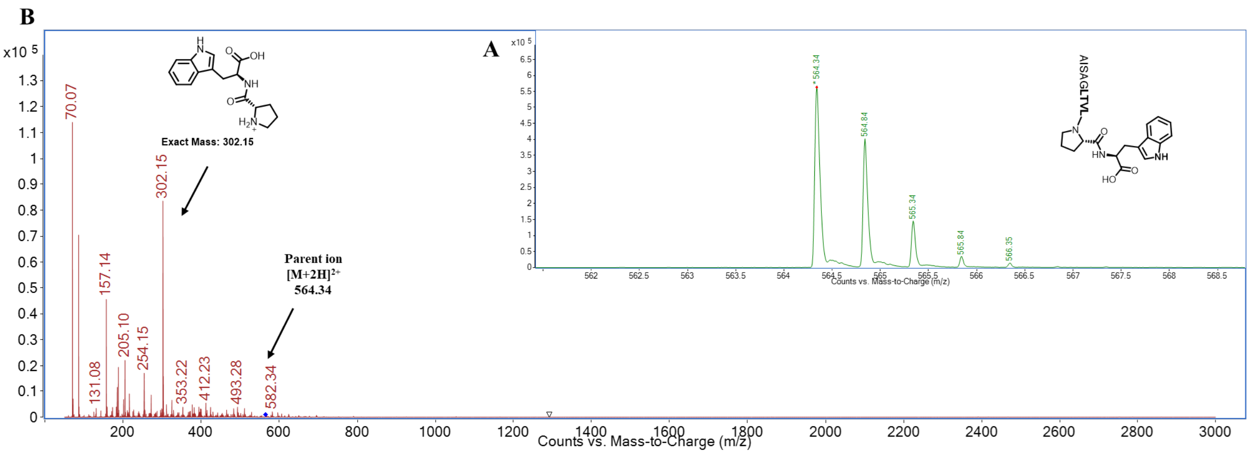

Figure S7: MS[1] and MS[2] spectra of the substrate peptide N-His$_6$-MBP-SrpE$^{(leader)}$-LTVLPW$^{(core)}$ after digestion with Glu-C. (**A**) The isotopic distribution of $[M+2H]^{2+}$ ions corresponding to the Glu-C digestion product. (**B**) MS[2] fragmentation spectra for the Glu-C digested product. The Pro-Trp daughter ion is structurally annotated. The $[M+2H]^{2+}$ parent ion is labeled.

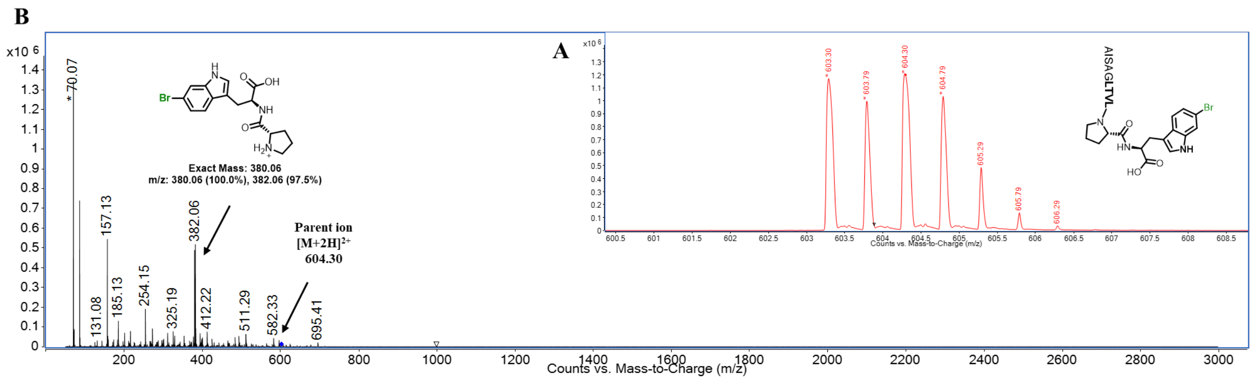

Figure S8: MS[1] and MS[2] spectra of brominated product N-His$_6$-MBP-SrpE$^{(leader)}$-LTVLPW$^{(core)}$ after digestion with Glu-C. (**A**) The isotopic distribution of $[M+2H]^{2+}$ ions corresponding to the Glu-C digestion product. (**B**) MS[2] fragmentation spectra for the Glu-C digested product. The Pro-Trp daughter ion is structurally annotated and denotes that the bromination is localized to the C-terminal Trp residue. The $[M+2H]^{2+}$ parent ion is labeled.

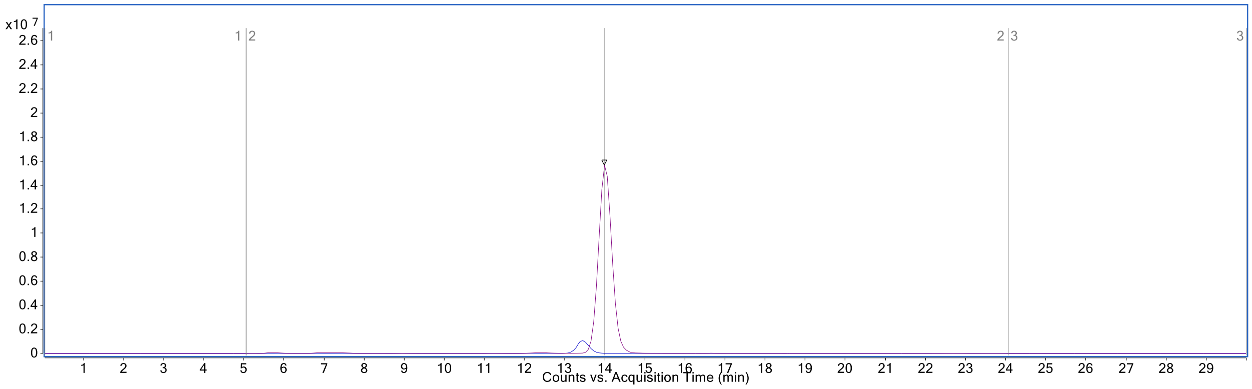

Figure S9: LC/MS analysis of the SrpI-catalyzed bromination of N-His$_6$-MBP-SrpE$^{(leader)}$-LTVLPW$^{(core)}$ peptide after digestion with Glu-C. Extracted ion chromatograms (EICs) corresponding to the unmodified substrate and the brominated product are illustrated. Area under these EICs was used to calculate the yield of the SrpI-catalyzed bromination reaction.

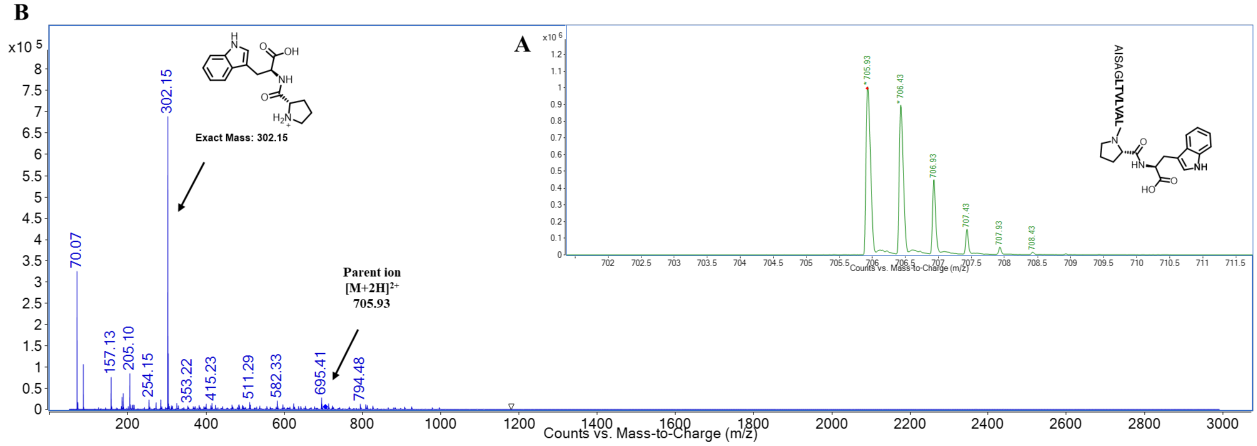

Figure S10: MS[1] and MS[2] spectra of the substrate peptide N-His$_6$-MBP-SrpE$^{(leader)}$-LTVLVALPW$^{(core)}$ after digestion with Glu-C. (**A**) The isotopic distribution of $[M+2H]^{2+}$ ions corresponding to the Glu-C digestion product. (**B**) MS[2] fragmentation spectra for the Glu-C digested product. The Pro-Trp daughter ion is structurally annotated. The $[M+2H]^{2+}$ parent ion is labeled.

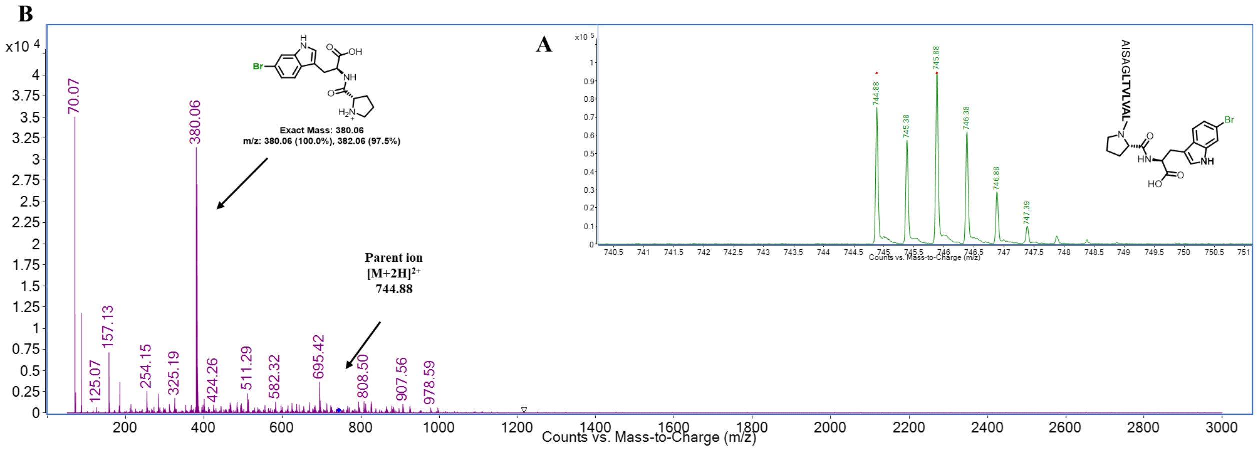

Figure S11: MS[1] and MS[2] spectra of brominated product N-His$_6$-MBP-SrpE$^{(leader)}$-LTVLVALPW$^{(core)}$ after digestion with Glu-C. (**A**) The isotopic distribution of $[M+2H]^{2+}$ ions corresponding to the Glu-C digestion product. (**B**) MS[2] fragmentation spectra for the Glu-C digested product. The Pro-Trp daughter ion is structurally annotated and denotes that the bromination is localized to the C-terminal Trp residue. The $[M+2H]^{2+}$ parent ion is labeled.

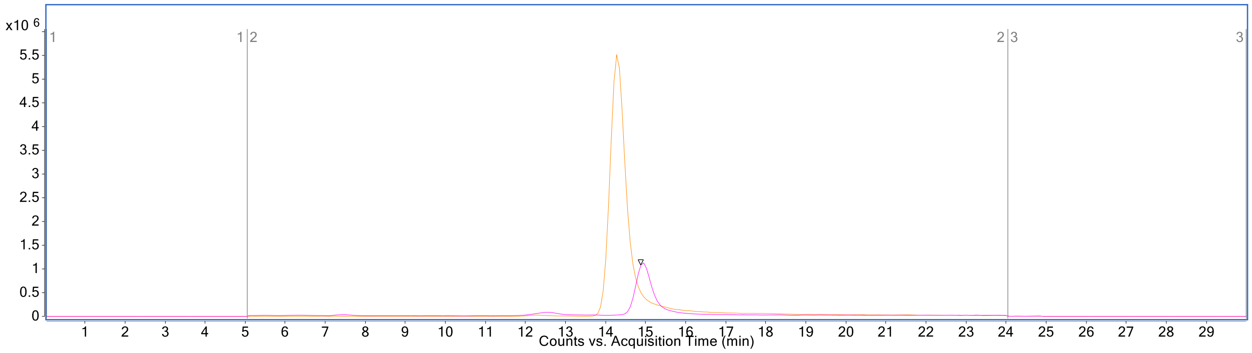

Figure S12: LC/MS analysis of the SrpI-catalyzed bromination of N-His$_6$-MBP-SrpE$^{(leader)}$-LTVLVALPW$^{(core)}$ peptide after digestion with Glu-C. Extracted ion chromatograms (EICs) corresponding to the unmodified substrate and the brominated product are illustrated. Area under these EICs was used to calculate the yield of the SrpI-catalyzed bromination reaction.

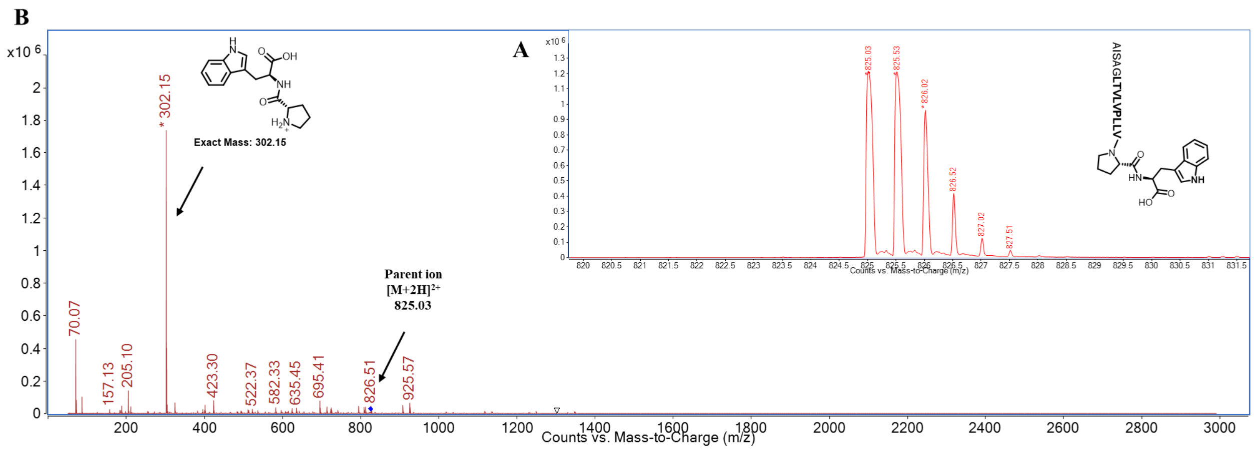

Figure S13: MS^1 and MS^2 spectra of the substrate peptide N-His$_6$-MBP-SrpE$^{(leader)}$-LTVLVPLLVPW$^{(core)}$ after digestion with Glu-C. (**A**) The isotopic distribution of $[M+2H]^{2+}$ ions corresponding to the Glu-C digestion product. (**B**) MS^2 fragmentation spectra for the Glu-C digested product. The Pro-Trp daughter ion is structurally annotated. The $[M+2H]^{2+}$ parent ion is labeled.

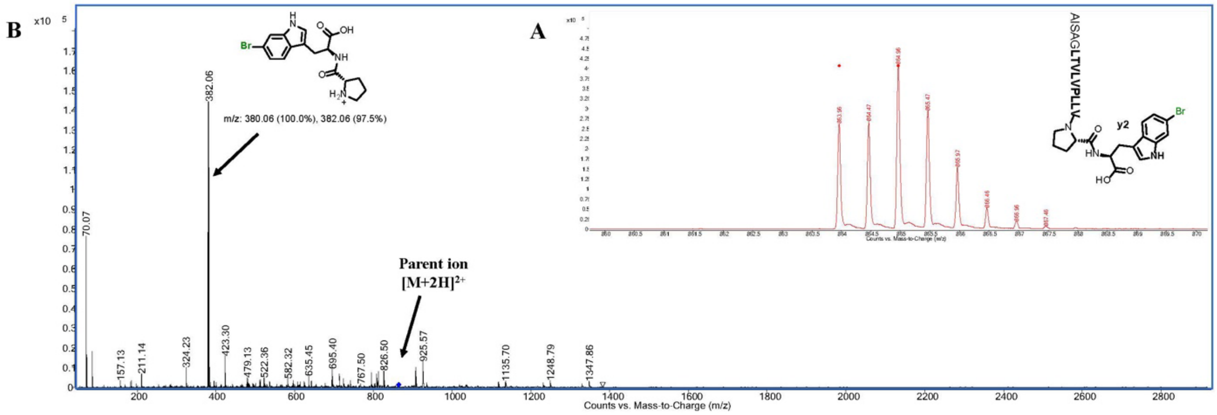

Figure S14: MS^1 and MS^2 spectra of brominated product N-His$_6$-MBP-SrpE$^{(leader)}$-LTVLVPLLVPW$^{(core)}$ after digestion with Glu-C. (**A**) The isotopic distribution of [M+2H]$^{2+}$ ions corresponding to the Glu-C digestion product. (**B**) MS^2 fragmentation spectra for the Glu-C digested product. The Pro-Trp daughter ion is structurally annotated and denotes that the bromination is localized to the C-terminal Trp residue. The [M+2H]$^{2+}$ parent ion is labeled.

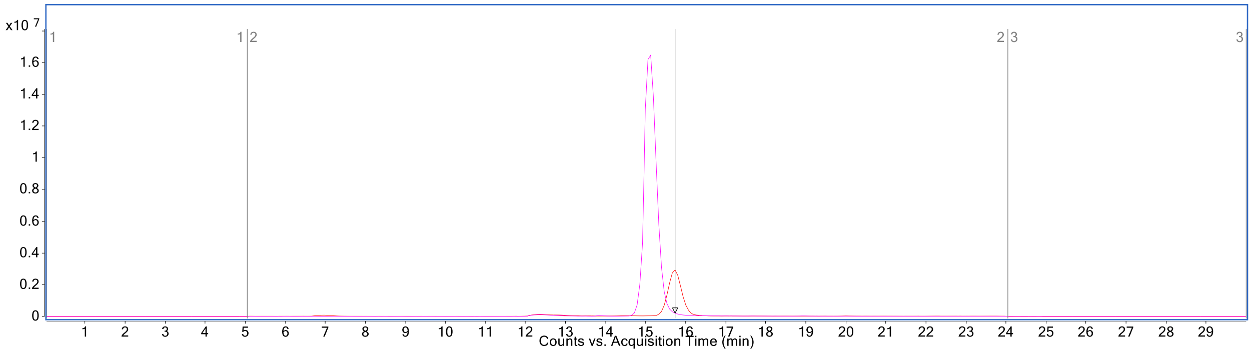

Figure S15: LC/MS analysis of the SrpI-catalyzed bromination of N-His$_6$-MBP-SrpE$^{(leader)}$-LTVLVPLLVPW$^{(core)}$ peptide after digestion with Glu-C. Extracted ion chromatograms (EICs) corresponding to the unmodified substrate and the brominated product. Area under these EICs was used to calculate the yield of the SrpI-catalyzed bromination reaction.

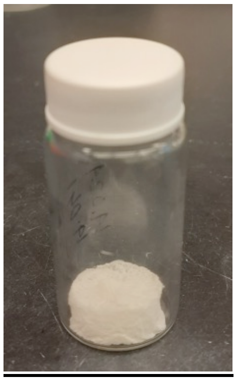

Figure S16: Lyophilized brominated peptide generated in preparative amounts by SrpI-catalyzed reaction.

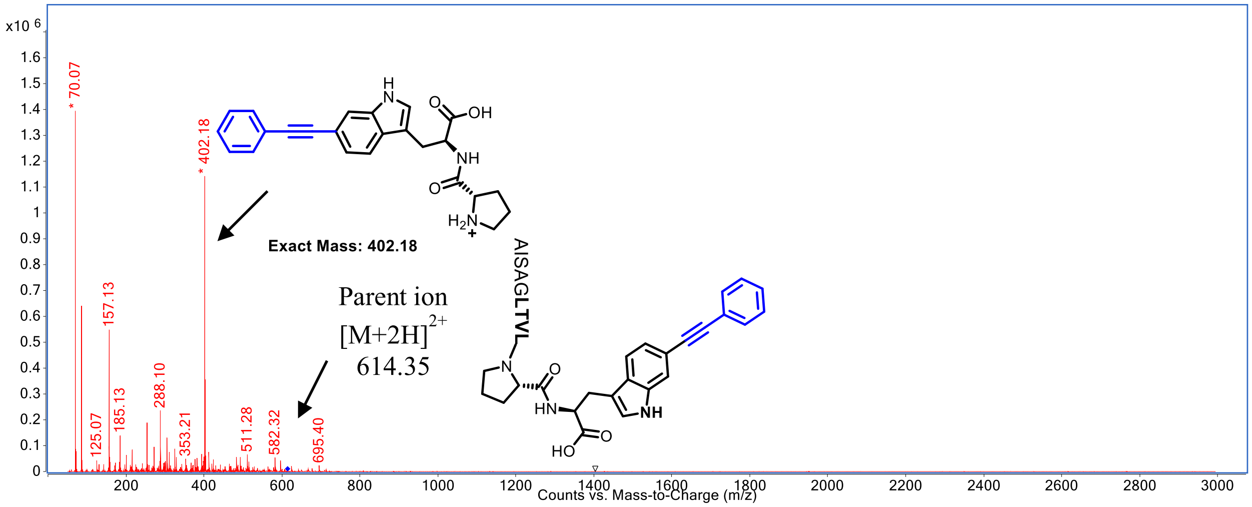

Figure S17: Characterization of the Sonogashira coupling of the alkyne **1** with the Glu-C digested product of N-His$_6$-MBP-SrpE$^{(leader)}$-LTVLPW$^{(core)}$-Br peptide. The characteristic Pro-Trp daughter ion is structurally annotated which demonstrates that the Sonogashira coupling has been affected upon the C-terminal Trp residue. The $[M+2H]^{2+}$ parent ion is labeled.

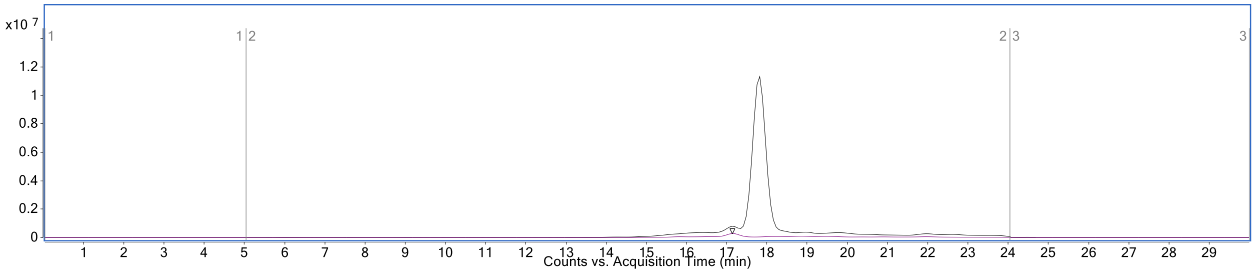

Figure S18: EICs corresponding to the Glu-C digested product derived from N-His_6-MBP-$SrpE^{(leader)}$-$LTVLPW^{(core)}$-Br peptide, and the peptide coupled to alkyne **1** using the copper-free Sonogashira reaction conditions developed in this study. The areas under the EICs were used to calculate the yield of the coupling reaction.

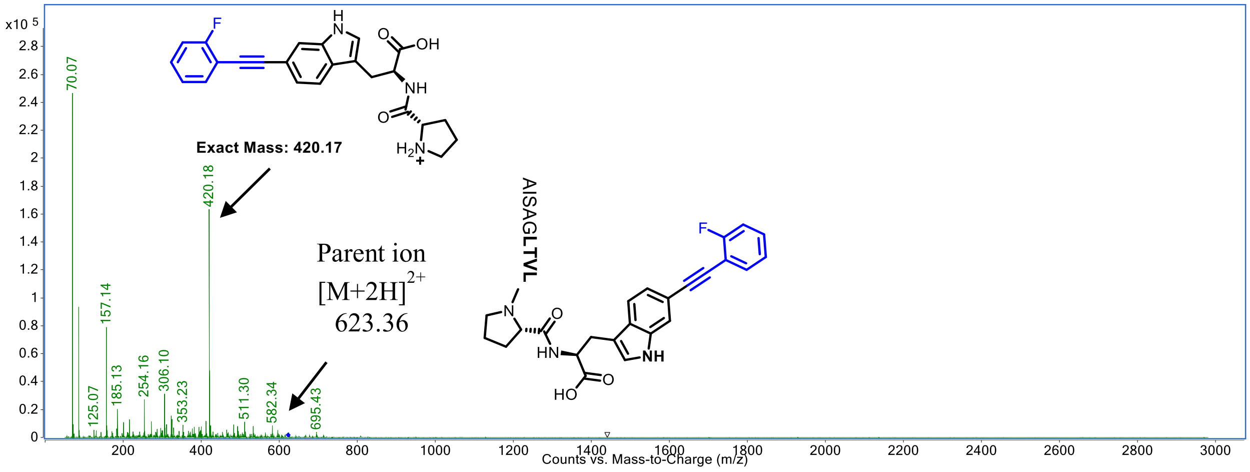

Figure S19: Characterization of the Sonogashira coupling of the alkyne **2** with the Glu-C digested product of N-His$_6$-MBP-SrpE$^{(leader)}$-LTVLPW$^{(core)}$-Br peptide. The characteristic Pro-Trp daughter ion is structurally annotated which demonstrates that the Sonogashira coupling has been affected upon the C-terminal Trp residue. The $[M+2H]^{2+}$ parent ion is labeled.

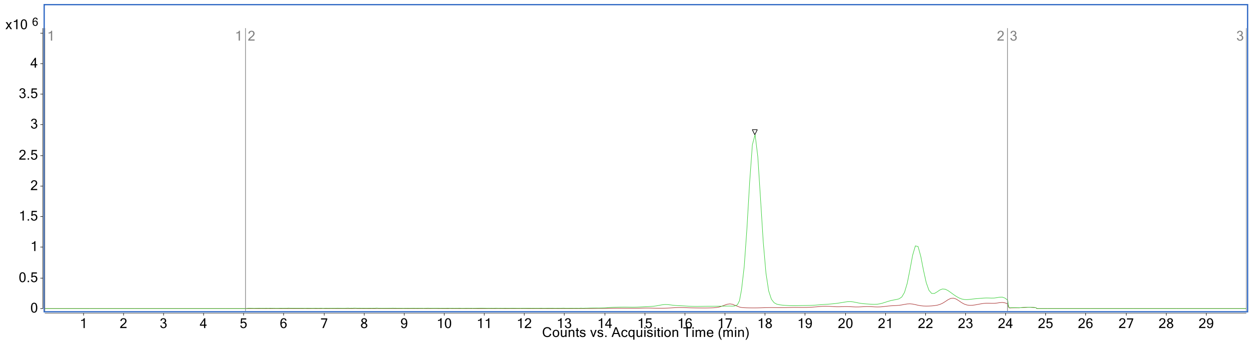

Figure S20: EICs corresponding to the Glu-C digested product derived from N-His$_6$-MBP-SrpE$^{(leader)}$-LTVLPW$^{(core)}$-Br peptide, and the peptide coupled to alkyne **2** using the copper-free Sonogashira reaction conditions developed in this study. The areas under the EICs were used to calculate the yield of the coupling reaction.

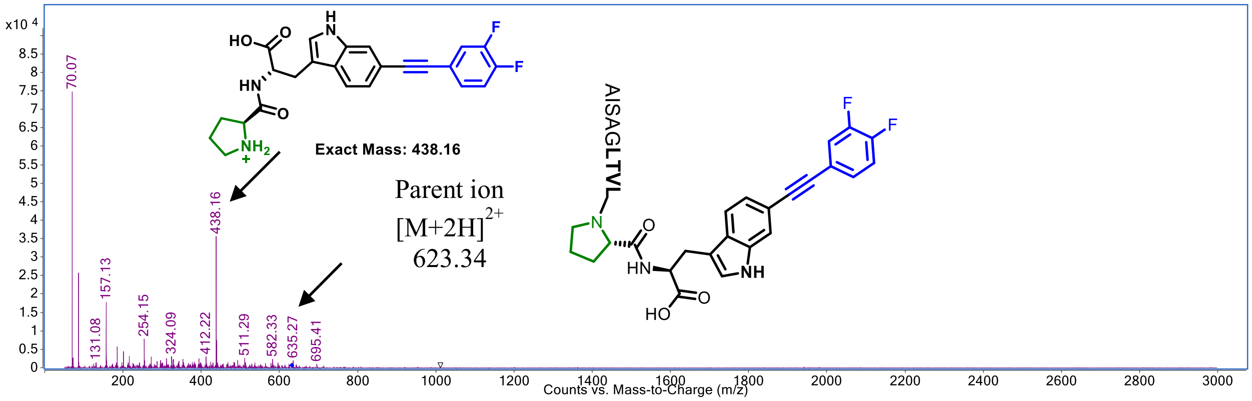

Figure S21: Characterization of the Sonogashira coupling of the alkyne **3** with the Glu-C digested product of N-His$_6$-MBP-SrpE$^{(leader)}$-LTVLPW$^{(core)}$-Br peptide. The characteristic Pro-Trp daughter ion is structurally annotated which demonstrates that the Sonogashira coupling has been affected upon the C-terminal Trp residue. The $[M+2H]^{2+}$ parent ion is labeled.

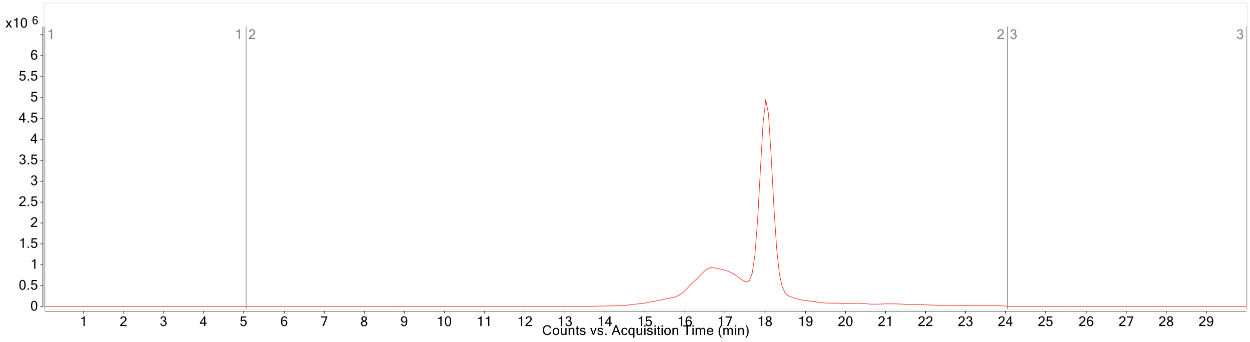

Figure S22: EICs corresponding to the Glu-C digested product derived from N-His$_6$-MBP-SrpE$^{(leader)}$-LTVLPW$^{(core)}$-Br peptide, and the peptide coupled to alkyne **3** using the copper-free Sonogashira reaction conditions developed in this study. The areas under the EICs were used to calculate the yield of the coupling reaction.

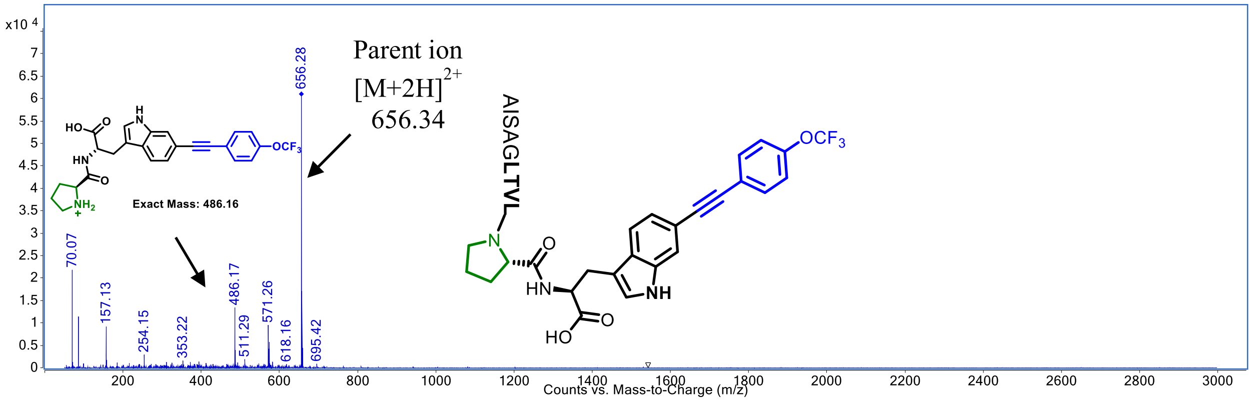

Figure S23: Characterization of the Sonogashira coupling of the alkyne **4** with the Glu-C digested product of N-His$_6$-MBP-SrpE$^{(leader)}$-LTVLPW$^{(core)}$-Br peptide. The characteristic Pro-Trp daughter ion is structurally annotated which demonstrates that the Sonogashira coupling has been affected upon the C-terminal Trp residue. The $[M+2H]^{2+}$ parent ion is labeled.

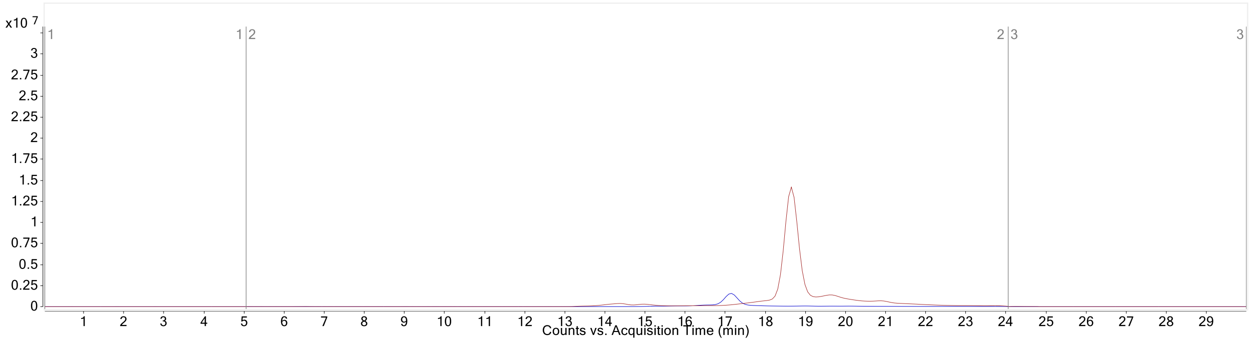

Figure S24: EICs corresponding to the Glu-C digested product derived from N-His$_6$-MBP-SrpE$^{(leader)}$-LTVLPW$^{(core)}$-Br peptide, and the peptide coupled to alkyne **4** using the copper-free Sonogashira reaction conditions developed in this study. The areas under the EICs were used to calculate the yield of the coupling reaction.

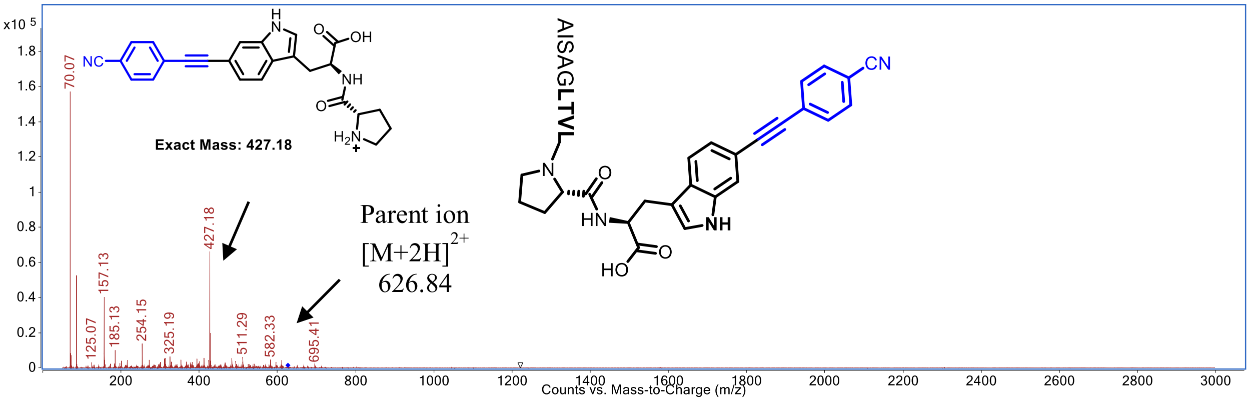

Figure S25: Characterization of the Sonogashira coupling of the alkyne **5** with the Glu-C digested product of N-His$_6$-MBP-SrpE$^{(leader)}$-LTVLPW$^{(core)}$-Br peptide. The characteristic Pro-Trp daughter ion is structurally annotated which demonstrates that the Sonogashira coupling has been affected upon the C-terminal Trp residue. The $[M+2H]^{2+}$ parent ion is labeled.

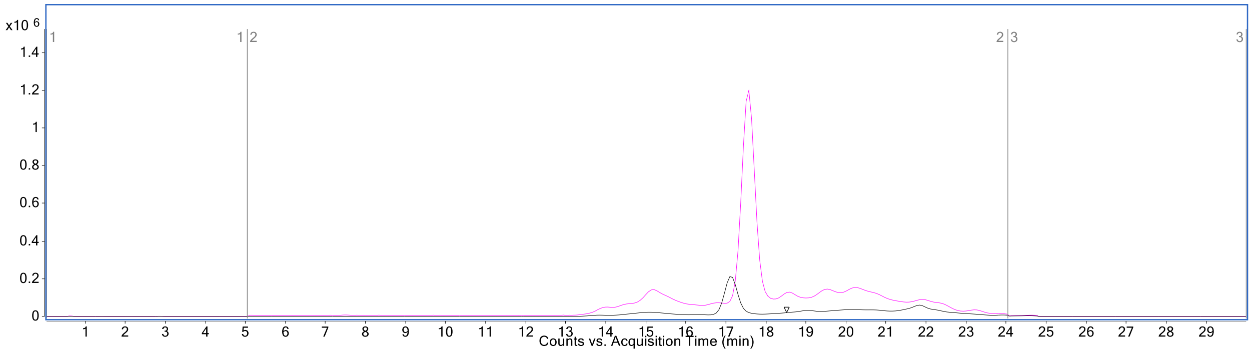

Figure S26: EICs corresponding to the Glu-C digested product derived from N-His$_6$-MBP-SrpE$^{(leader)}$-LTVLPW$^{(core)}$-Br peptide, and the peptide coupled to alkyne **5** using the copper-free Sonogashira reaction conditions developed in this study. The areas under the EICs were used to calculate the yield of the coupling reaction.

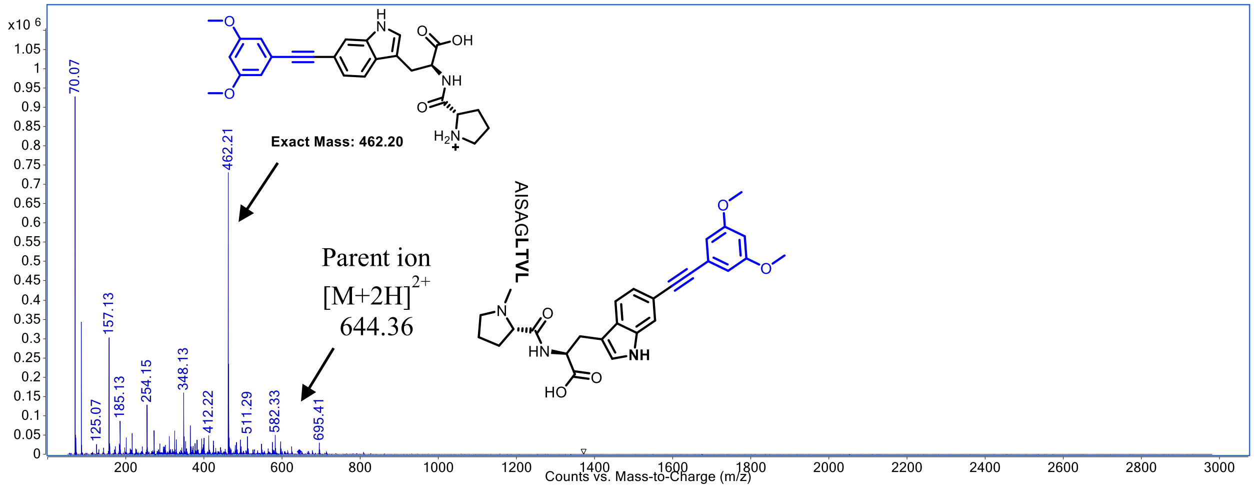

Figure S27: Characterization of the Sonogashira coupling of the alkyne **6** with the Glu-C digested product of N-His$_6$-MBP-SrpE$^{(leader)}$-LTVLPW$^{(core)}$-Br peptide. The characteristic Pro-Trp daughter ion is structurally annotated which demonstrates that the Sonogashira coupling has been affected upon the C-terminal Trp residue. The $[M+2H]^{2+}$ parent ion is labeled.

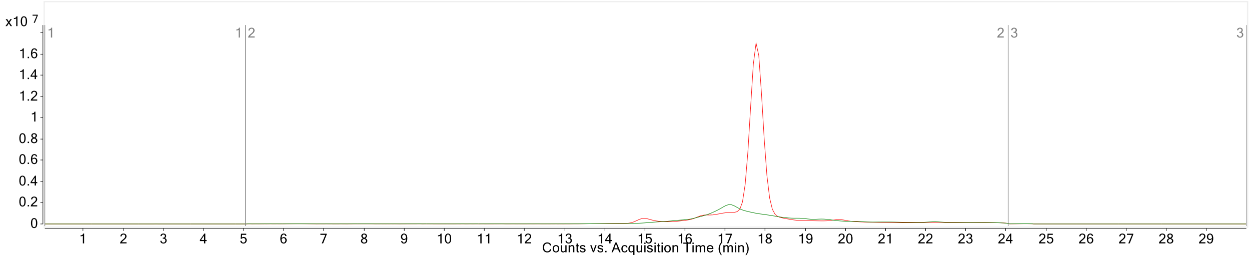

Figure S28: EICs corresponding to the Glu-C digested product derived from N-His$_6$-MBP-SrpE$^{(leader)}$-LTVLPW$^{(core)}$-Br peptide, and the peptide coupled to alkyne **6** using the copper-free Sonogashira reaction conditions developed in this study. The areas under the EICs were used to calculate the yield of the coupling reaction.

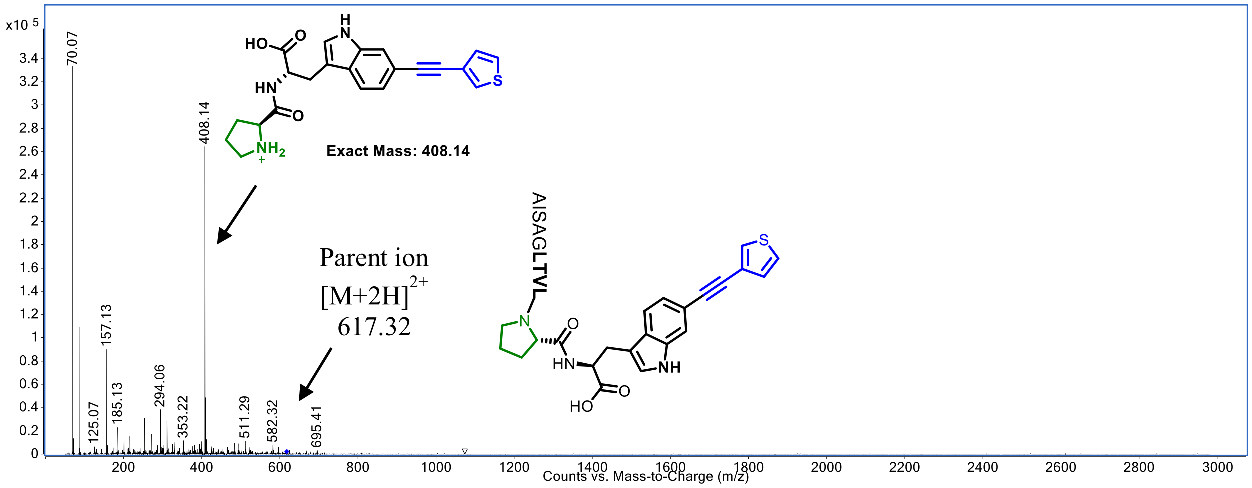

Figure S29: Characterization of the Sonogashira coupling of the alkyne **7** with the Glu-C digested product of N-His$_6$-MBP-SrpE$^{(leader)}$-LTVLPW$^{(core)}$-Br peptide. The characteristic Pro-Trp daughter ion is structurally annotated which demonstrates that the Sonogashira coupling has been affected upon the C-terminal Trp residue. The $[M+2H]^{2+}$ parent ion is labeled.

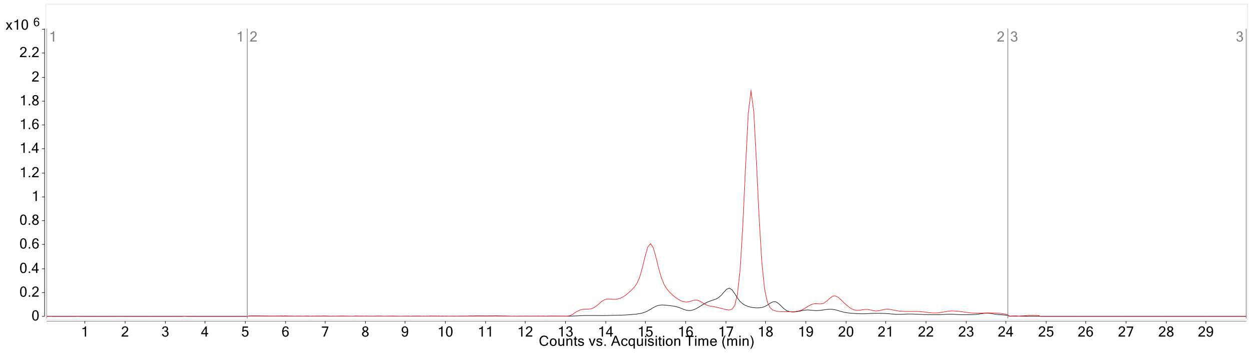

Figure S30: EICs corresponding to the Glu-C digested product derived from N-His$_6$-MBP-SrpE$^{(leader)}$-LTVLPW$^{(core)}$-Br peptide, and the peptide coupled to alkyne **7** using the copper-free Sonogashira reaction conditions developed in this study. The areas under the EICs were used to calculate the yield of the coupling reaction.

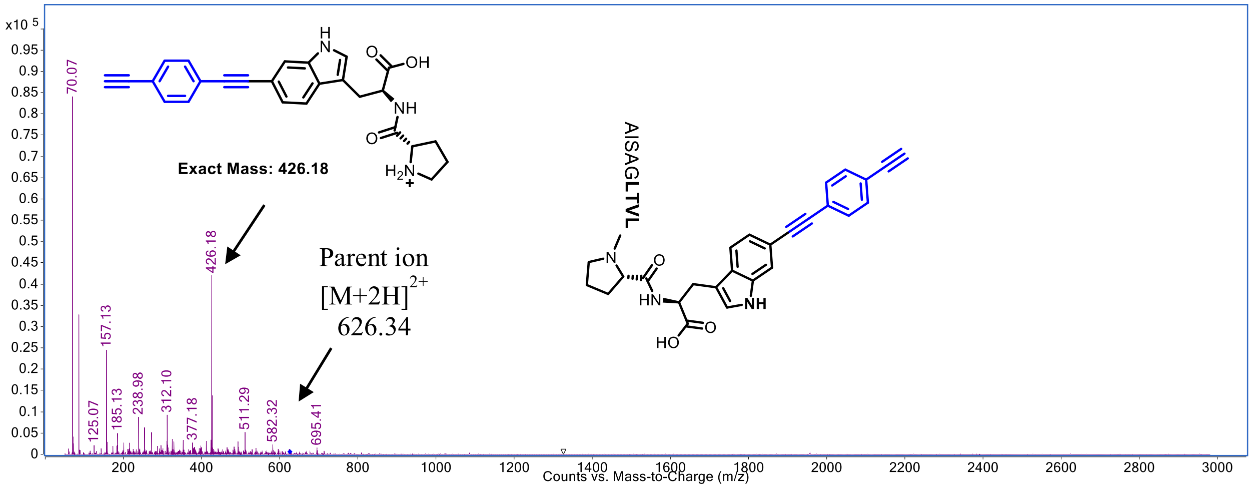

Figure S31: Characterization of the Sonogashira coupling of the alkyne **8** with the Glu-C digested product of N-His$_6$-MBP-SrpE$^{(leader)}$-LTVLPW$^{(core)}$-Br peptide. The characteristic Pro-Trp daughter ion is structurally annotated which demonstrates that the Sonogashira coupling has been affected upon the C-terminal Trp residue. The $[M+2H]^{2+}$ parent ion is labeled.

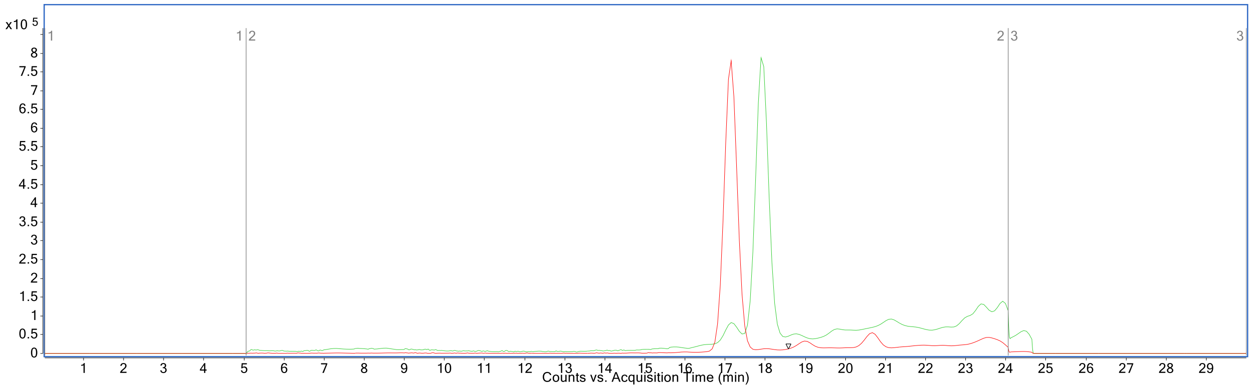

Figure S32: EICs corresponding to the Glu-C digested product derived from N-His$_6$-MBP-SrpE$^{(leader)}$-LTVLPW$^{(core)}$-Br peptide, and the peptide coupled to alkyne **8** using the copper-free Sonogashira reaction conditions developed in this study. The areas under the EICs were used to calculate the yield of the coupling reaction.

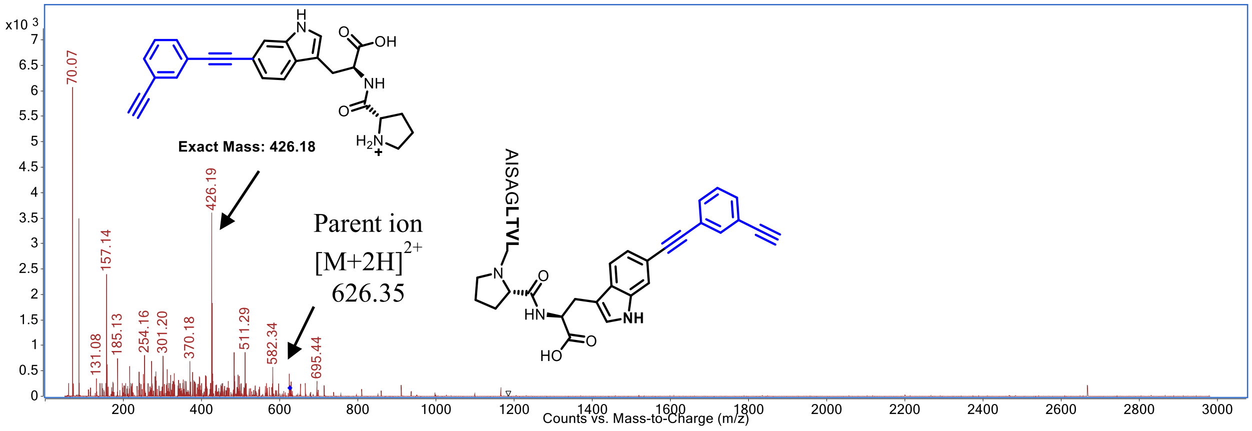

Figure S33: Characterization of the Sonogashira coupling of the alkyne **9** with the Glu-C digested product of N-His$_6$-MBP-SrpE$^{(leader)}$-LTVLPW$^{(core)}$-Br peptide. The characteristic Pro-Trp daughter ion is structurally annotated which demonstrates that the Sonogashira coupling has been affected upon the C-terminal Trp residue. The $[M+2H]^{2+}$ parent ion is labeled.

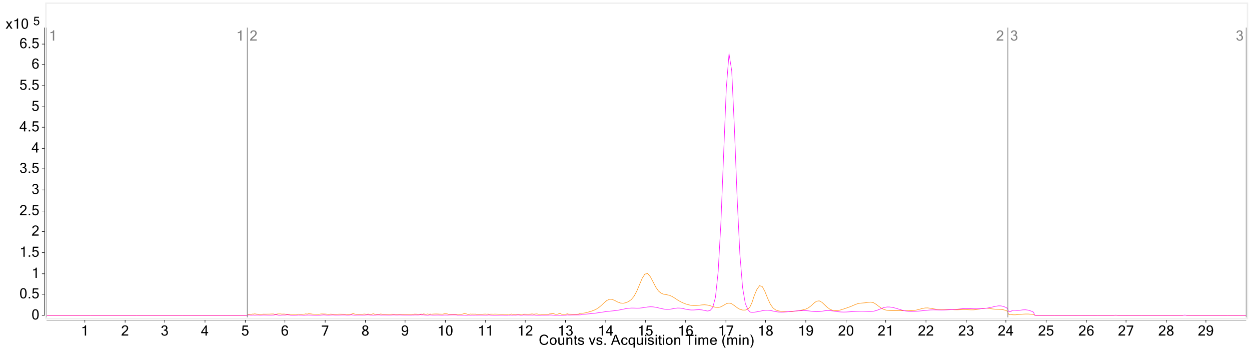

Figure S34: EICs corresponding to the Glu-C digested product derived from N-His$_6$-MBP-SrpE$^{(leader)}$-LTVLPW$^{(core)}$-Br peptide, and the peptide coupled to alkyne **9** using the copper-free Sonogashira reaction conditions developed in this study. The areas under the EICs were used to calculate the yield of the coupling reaction.

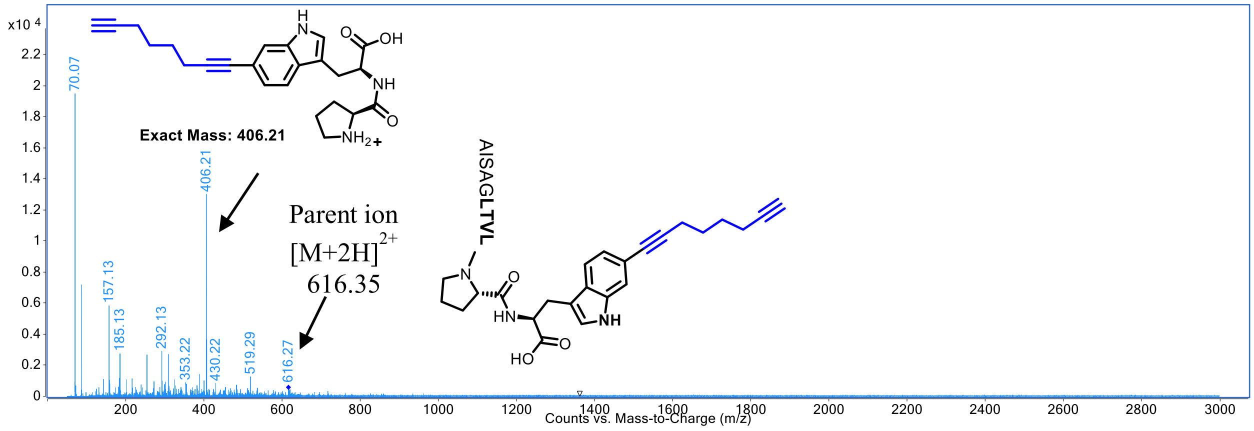

Figure S35: Characterization of the Sonogashira coupling of the alkyne **10** with the Glu-C digested product of N-His$_6$-MBP-SrpE$^{(leader)}$-LTVLPW$^{(core)}$-Br peptide. The characteristic Pro-Trp daughter ion is structurally annotated which demonstrates that the Sonogashira coupling has been affected upon the C-terminal Trp residue. The $[M+2H]^{2+}$ parent ion is labeled.

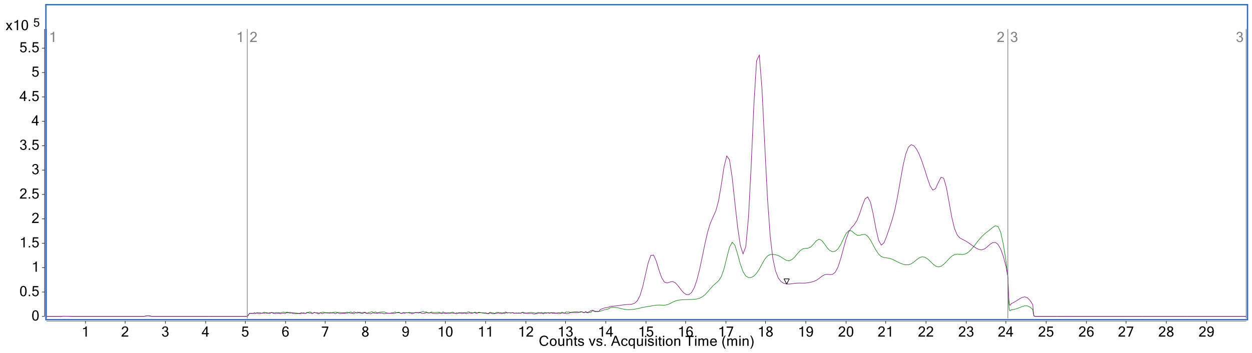

Figure S36: EICs corresponding to the Glu-C digested product derived from N-His$_6$-MBP-SrpE$^{(leader)}$-LTVLPW$^{(core)}$-Br peptide, and the peptide coupled to alkyne **10** using the copper-free Sonogashira reaction conditions developed in this study. The areas under the EICs were used to calculate the yield of the coupling reaction.

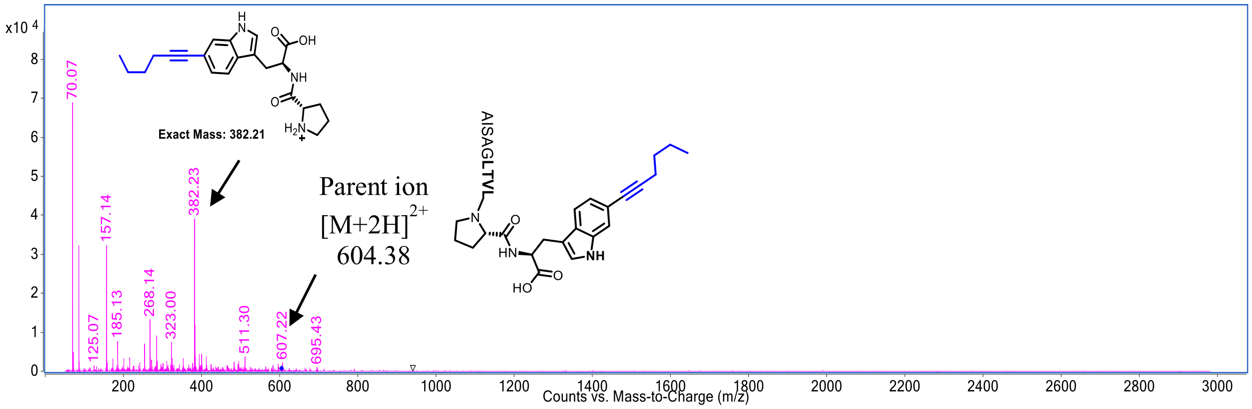

Figure S37: Characterization of the Sonogashira coupling of the alkyne **11** with the Glu-C digested product of N-His$_6$-MBP-SrpE$^{(leader)}$-LTVLPW$^{(core)}$-Br peptide. The characteristic Pro-Trp daughter ion is structurally annotated which demonstrates that the Sonogashira coupling has been affected upon the C-terminal Trp residue. The $[M+2H]^{2+}$ parent ion is labeled.

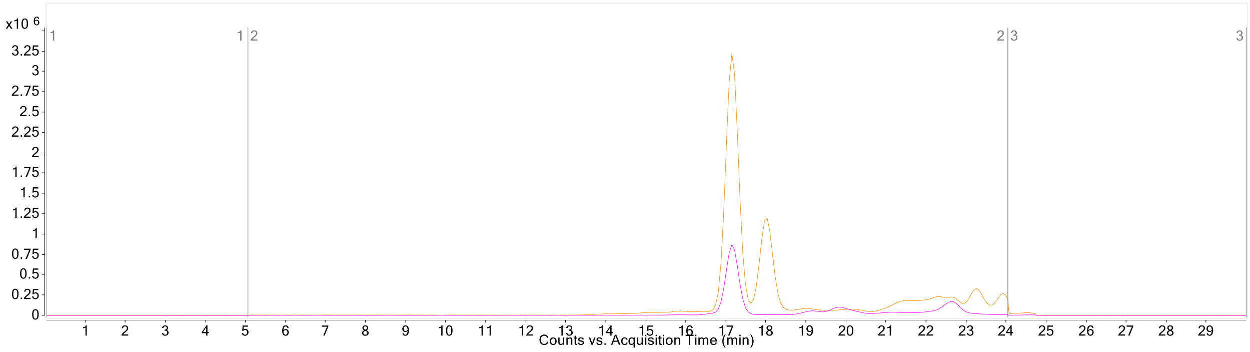

Figure S38: EICs corresponding to the Glu-C digested product derived from N-His$_6$-MBP-SrpE$^{(leader)}$-LTVLPW$^{(core)}$-Br peptide, and the peptide coupled to alkyne **11** using the copper-free Sonogashira reaction conditions developed in this study. The areas under the EICs were used to calculate the yield of the coupling reaction.

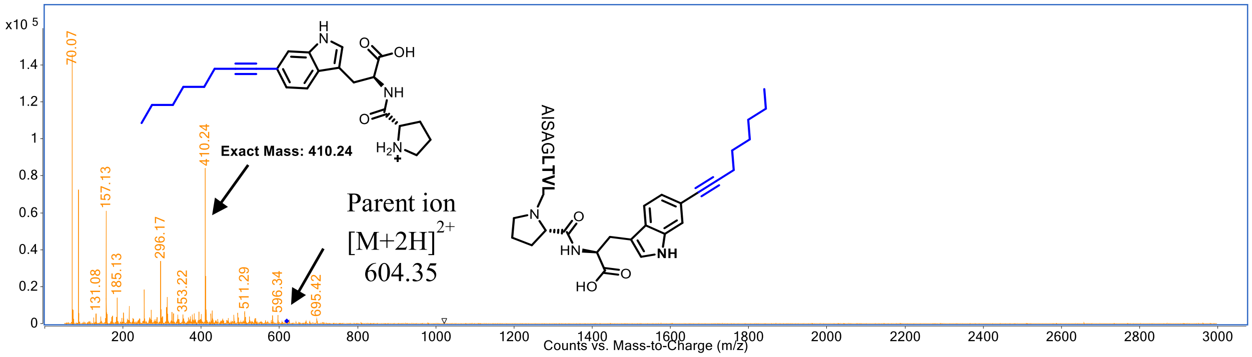

Figure S39: Characterization of the Sonogashira coupling of the alkyne **12** with the Glu-C digested product of N-His$_6$-MBP-SrpE$^{(leader)}$-LTVLPW$^{(core)}$-Br peptide. The characteristic Pro-Trp daughter ion is structurally annotated which demonstrates that the Sonogashira coupling has been affected upon the C-terminal Trp residue. The $[M+2H]^{2+}$ parent ion is labeled.

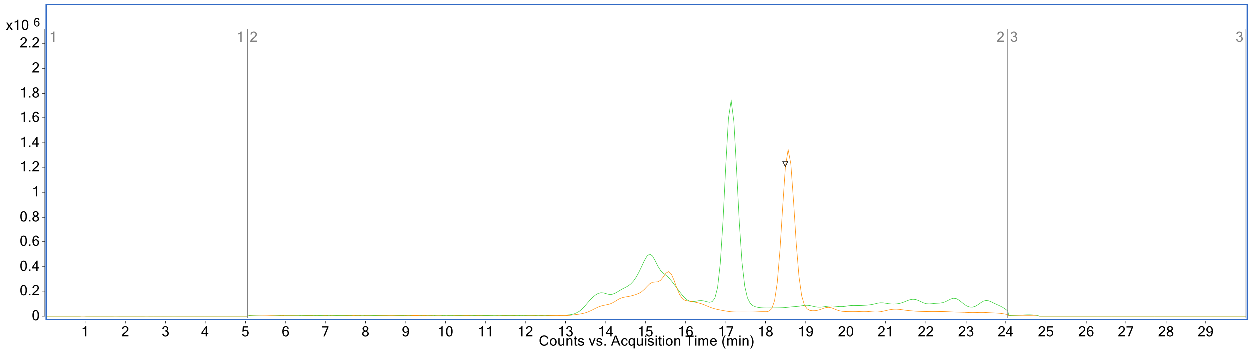

Figure S40: EICs corresponding to the Glu-C digested product derived from N-His$_6$-MBP-SrpE$^{(leader)}$-LTVLPW$^{(core)}$-Br peptide, and the peptide coupled to alkyne **12** using the copper-free Sonogashira reaction conditions developed in this study. The areas under the EICs were used to calculate the yield of the coupling reaction.

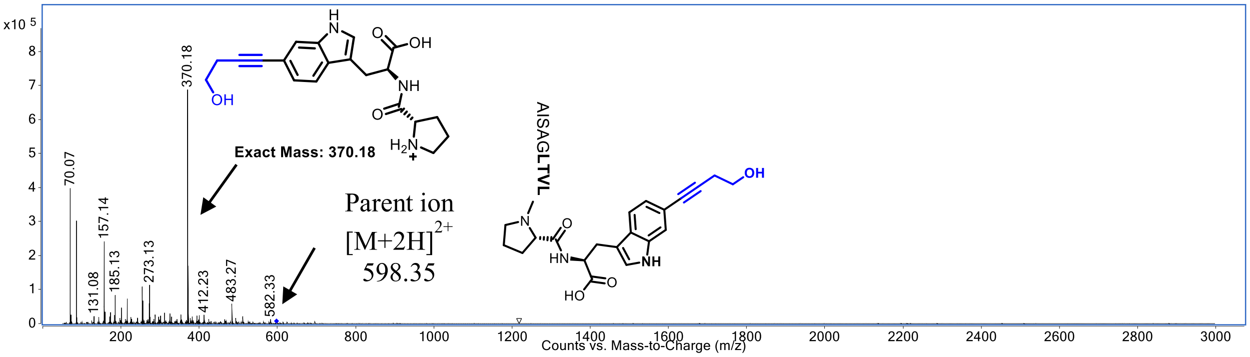

Figure S41: Characterization of the Sonogashira coupling of the alkyne **13** with the Glu-C digested product of N-His$_6$-MBP-SrpE$^{(leader)}$-LTVLPW$^{(core)}$-Br peptide. The characteristic Pro-Trp daughter ion is structurally annotated which demonstrates that the Sonogashira coupling has been affected upon the C-terminal Trp residue. The $[M+2H]^{2+}$ parent ion is labeled.

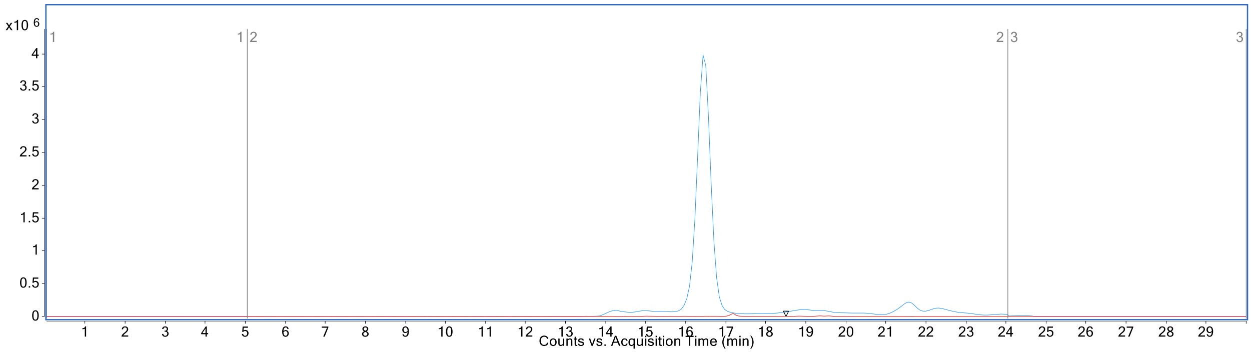

Figure S42: EICs corresponding to the Glu-C digested product derived from N-His$_6$-MBP-SrpE$^{(leader)}$-LTVLPW$^{(core)}$-Br peptide, and the peptide coupled to alkyne **13** using the copper-free Sonogashira reaction conditions developed in this study. The areas under the EICs were used to calculate the yield of the coupling reaction.

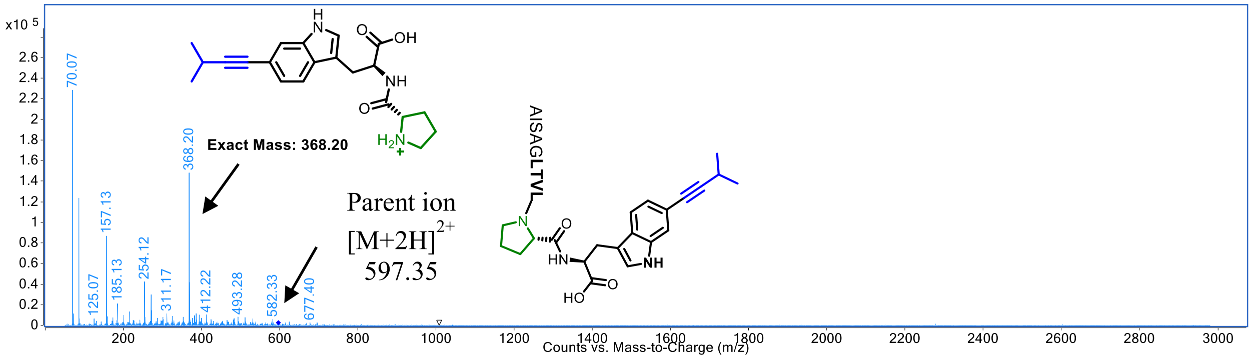

Figure S43: Characterization of the Sonogashira coupling of the alkyne **14** with the Glu-C digested product of N-His$_6$-MBP-SrpE$^{(leader)}$-LTVLPW$^{(core)}$-Br peptide. The characteristic Pro-Trp daughter ion is structurally annotated which demonstrates that the Sonogashira coupling has been affected upon the C-terminal Trp residue. The $[M+2H]^{2+}$ parent ion is labeled.

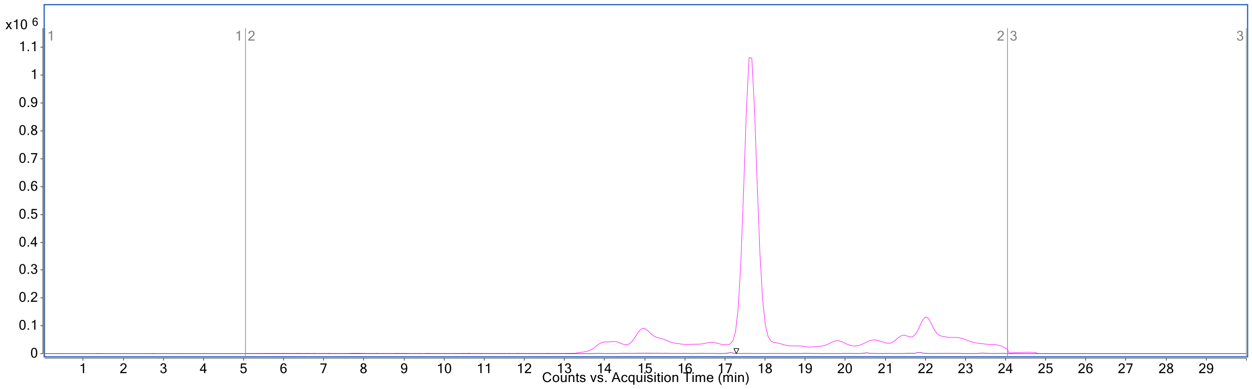

Figure S44: EICs corresponding to the Glu-C digested product derived from N-His$_6$-MBP-SrpE$^{(leader)}$-LTVLPW$^{(core)}$-Br peptide, and the peptide coupled to alkyne **14** using the copper-free Sonogashira reaction conditions developed in this study. The areas under the EICs were used to calculate the yield of the coupling reaction.

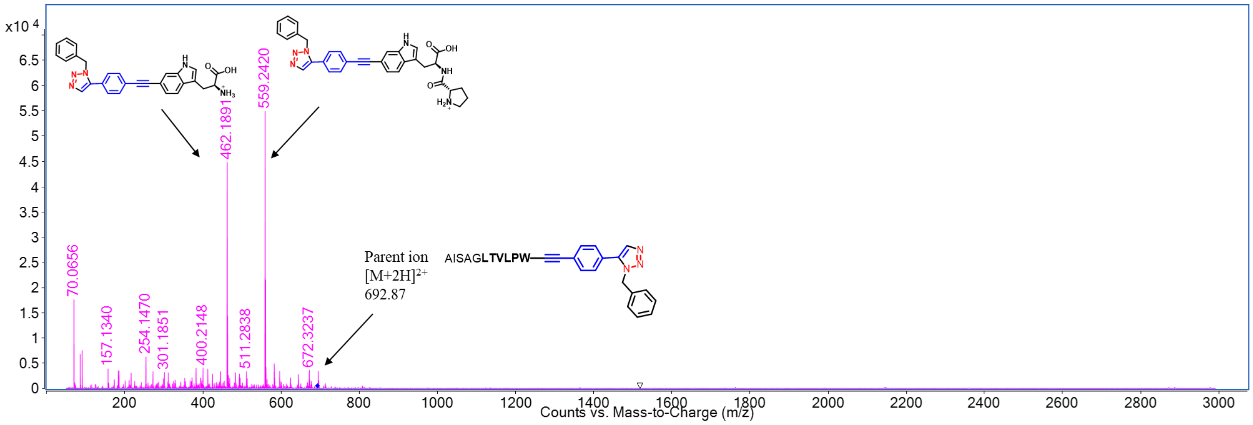

Figure S45: Characterization of the CuAAC product obtained using the peptidic alkyne **15** and benzyl azide as substrates. The characteristic daughter ions are structurally annotated. The $[M+2H]^{2+}$ parent ion is labeled with a blue diamond.

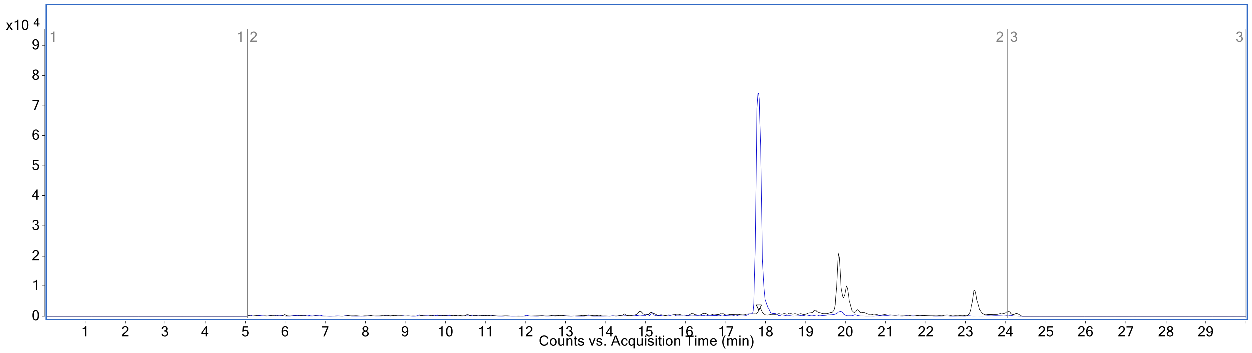

Figure S46: Overlaid EICs corresponding to the substrate peptidic alkyne **15** and the CuAAC product obtained upon reaction with benzyl azide. The areas under the EICs were used to calculate the yield of the coupling reaction. Only the triazole peptide product was observed; left over **15** was not detected.

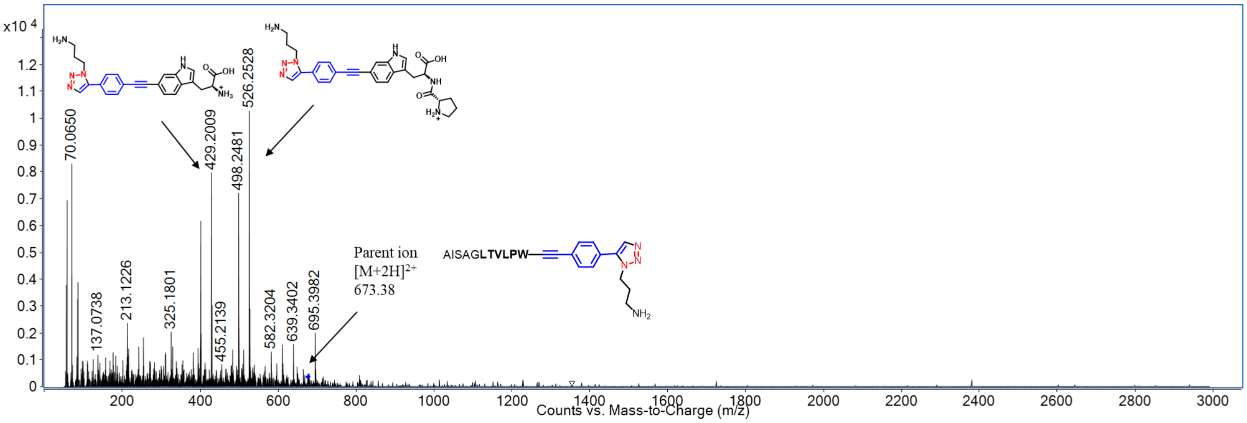

Figure S47: Characterization of the CuAAC product obtained using the peptidic alkyne **15** and 3-azido-1-propanamine as substrates. The characteristic daughter ions are structurally annotated. The $[M+2H]^{2+}$ parent ion is labeled with a blue diamond.

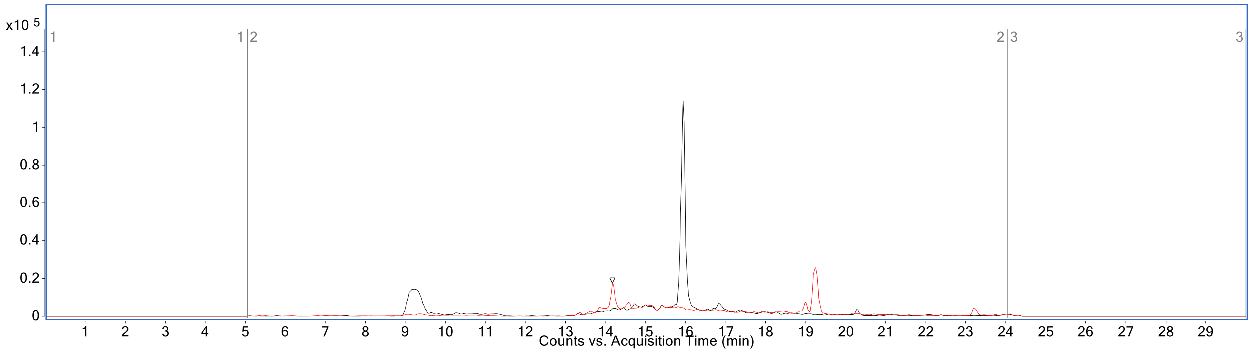

Figure S48: Overlaid EICs corresponding to the substrate peptidic alkyne **15** and the CuAAC product obtained upon reaction with 3-azido-1-propanamine. The areas under the EICs were used to calculate the yield of the coupling reaction. Only the triazole peptide product was observed; left over **15** was not detected.

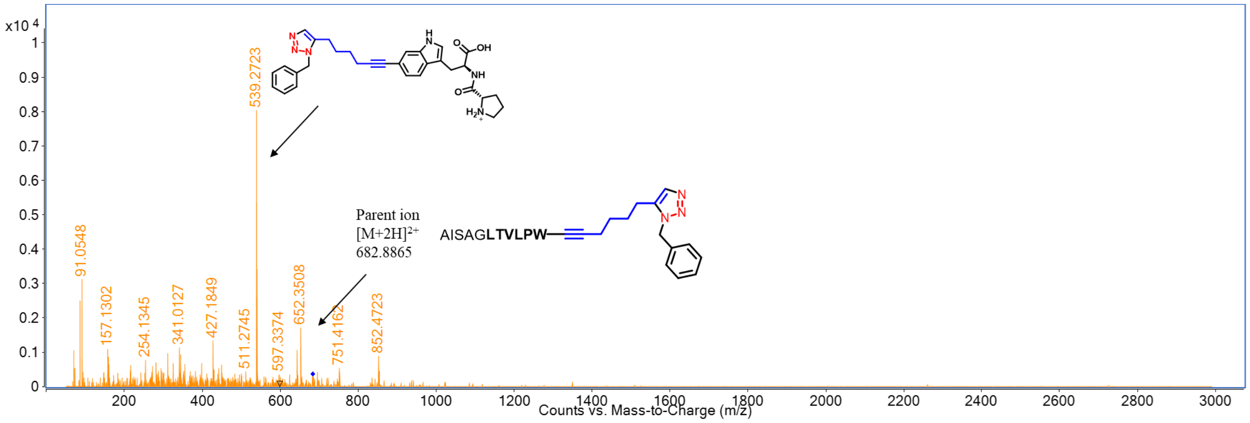

Figure S49: Characterization of the CuAAC product obtained using the peptidic alkyne **16** and benzyl azide as substrates. The characteristic daughter ions are structurally annotated. The $[M+2H]^{2+}$ parent ion is labeled with a blue diamond.

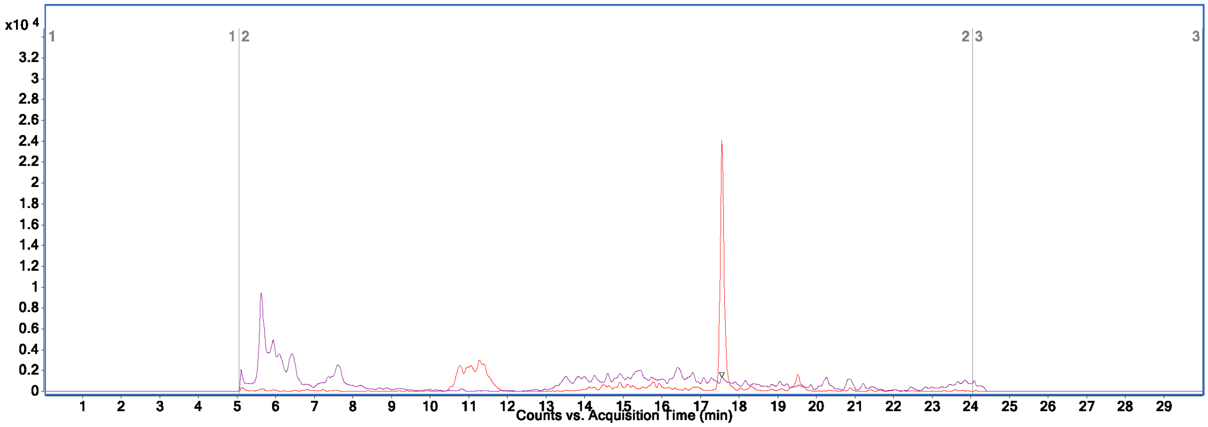

Figure S50: Overlaid EICs corresponding to the substrate peptidic alkyne **16** and the CuAAC product obtained upon reaction with benzyl azide. The areas under the EICs were used to calculate the yield of the coupling reaction. Only the triazole peptide product was observed; left over **16** was not detected.

Supplementary References

1. Nguyen, N. A.; Vidya, F. N. U.; Yennawar, N. H.; Wu, H.; McShan, A. C.; Agarwal, V., Disordered regions in proteusin peptides guide post-translational modification by a flavin-dependent RiPP brominase. *Nature Communications* **2024,** *15* (1), 1265.
2. Anderson, K. W.; Buchwald, S. L., General catalysts for the Suzuki–Miyaura and Sonogashira coupling reactions of aryl chlorides and for the coupling of challenging substrate combinations in water. *Angewandte Chemie International Edition* **2005,** *44* (38), 6173-6177.
